# Community gardens and their effects on diet, health, psychosocial and community outcomes: a systematic review

**DOI:** 10.1186/s12889-022-13591-1

**Published:** 2022-06-23

**Authors:** Clare Hume, Jessica A. Grieger, Anna Kalamkarian, Katina D’Onise, Lisa G. Smithers

**Affiliations:** 1 School of Public Health, University of Adelaide, Adelaide, 5000 Australia; 2 Adelaide Medical School, North Terrace, University of Adelaide, Adelaide, SA 5000 Australia; 3 Robinson Research Institute, University of Adelaide, North Adelaide, SA 5006 Australia; 4 Wellbeing SA, Citicentre, Hindmarsh Square, Adelaide, SA 5000 Australia; 5 School of Health and Society, University of Wollongong, Wollongong, NSW 2522 Australia

**Keywords:** Community gardens, Diet, Physical activity, Psychosocial, Health, Background

## Abstract

**Background:**

We systematically reviewed the effects of community gardens on physical and psychosocial health, health behaviors and community outcomes.

**Methods:**

Quantitative studies that examined associations of health, psychosocial or community outcomes with community gardens were included in the review. Studies up to December 2020 were captured from searches of Medline, Web of Science, PsycInfo, EBSCOHost and CAB Abstracts. Data were extracted and study quality including risk of bias was examined.

**Results:**

There were 53 studies that met the inclusion criteria. Studies examining associations between community gardens and nutrition or food security were most frequently reported (*k* = 23). Other factors examined for associations with community gardens were health (*k* = 16), psychosocial (*k* = 16) and community outcomes (*k* = 7). Effects appeared positive for fruit and vegetable intake, some psychosocial and community outcomes, but mixed for physical health outcomes. Evidence quality overall was low.

**Conclusions:**

Community gardening was associated with higher fruit and vegetable intake, positive psychosocial and community outcomes, but poor evidence quality suggests the effects of community gardening may be overestimated.

**Supplementary Information:**

The online version contains supplementary material available at 10.1186/s12889-022-13591-1.

## Background

Poor diets and physical inactivity are prominent contributors to chronic diseases [1]. Dietary risks factors are thought to directly contribute 5–14% to all death and disability in high-income countries like Australia, the United Kingdom, Canada and the United States [2]. Interventions to improve diet and physical activity have become an important focus for public health and for governments, with environmental factors receiving attention [3]. Interventions that involve environmental activities such as gardening are thought to have several health benefits including physical, mental and psychosocial outcomes [4].

Conceptual models such as the one proposed by Lovell and colleagues [5] suggest several health and community benefits of participating in gardening, particularly in communal spaces. Gardening is a physically active pastime [6] and may also address food and nutrition-related factors, through fruit and vegetable production and consumption [7]. Gardening also encourages experiences in nature which may have effects independent of other health behaviors such as stress reduction [8]. Participation in community gardening activities may encourage social interactions and the development of social support networks, as well as broader community-level components such as social cohesiveness and neighborhood attachment [5]. Thus, gardening in communal spaces may be useful for chronic disease treatment and prevention by targeting multiple health behaviors, but also concurrently addressing individual-level psychosocial outcomes such as social isolation, mental health and general wellbeing. Community gardens fall under the umbrella of ‘urban agriculture’, which incorporates both domestic or home-based gardens, as well as gardens open to community members for the purposes of growing, cultivating and taking care of plants and flowers for non-commercial outcomes [4]. The current review will specifically focus on the latter type of garden, spaces open to the general public or community.

Previous publications have reviewed the evidence primarily for the effects of community gardening on food and nutrition-related outcomes. Garcia and colleagues [9] reviewed studies examining urban gardens and food and nutrition outcomes among adults, with evidence of positive outcomes on fruit and vegetable consumption, access to healthy foods, as well as improved food perceptions such as the value of organic production and cooking. Importantly, that review was limited to studies among adults, food and nutrition outcomes, and studies of home-based gardens rather than community gardens. McCormack and colleagues [10] reached similar conclusions from their review of community gardens studies conducted only in the United States, as well as methodological issues identified in the studies reviewed. Such findings were echoed by Audate et al. in their scoping review of urban agriculture and its effects on health, wellbeing, food security and social capital [11]. Recently, Kunpeuk et al. [12] conducted a meta-analysis on the health and nutrition-related outcomes associated with community gardening, which suggested a positive effect of community garden participation on body mass index (BMI).

While there have been reviews on community gardening, most past reviews only consider nutrition-related outcomes in isolation from other, broader health factors, or behavioral and psychosocial outcomes. The potential for wider neighborhood-level benefits of community gardens have been understudied. By bringing together information on multiple outcomes we hoped to establish a comprehensive view of the evidence on community gardens that is broader in scope. Therefore, the aim of this work is to systematically review the evidence on effects of community gardens for effects on the following outcomes:

1. Food consumption, with particular attention to vegetable and fruit intake
2. Health outcomes, with particular attention to physical activity
3. Psychosocial measures, such as (but not limited to) social isolation, mental health and wellbeing
4. Community sentiment, such as (but not limited to) social cohesiveness

Additionally, we aimed to collate information on the characteristics of people who use community gardens and whether the effects of community gardens on outcomes might differ according to location (urban, regional, remote) or socioeconomic position.

## Materials and methods

The methods were undertaken according to a pre-written protocol which is available from the authors upon request. The review was undertaken using standard systematic review methodology following the Cochrane Collaboration methods and is reported according to the PRISMA guidelines [13].

### Search strategy

We searched Medline via PubMed platform, Web of Science, PsycINFO, EBSCOhost and CAB Abstracts from inception until 4^th^ December 2020. To capture literature across all the key outcome areas, the search strategy was deliberately broad in scope, covering databases from health, psychology and sociology. The search strategy was tailored to each database and search terms were pilot tested. MeSH terms and keywords from relevant articles were reviewed to design searches most likely to identify relevant articles. When possible, searches were limited to articles published in English and to humans, and searches were not limited by date or by setting (e.g. high and low-middle-income countries were eligible). In addition to the search strategy described above, we reviewed the reference lists of systematic reviews in this field for potentially relevant studies. The search strategies for each database are included in Supplementary Table 1.

### Eligibility and PICO (Participants, Intervention, Comparator and Outcomes) criteria

Studies that make inferences about community gardens were included. Quantitative studies were prioritized for evaluation; qualitative studies were excluded from the review unless they also reported quantitative data. Evidence from randomized controlled trials (RCTs) were considered separately from observational studies and case studies were excluded. Ecological studies were eligible for inclusion as implementation of community gardens may often occur at the higher community (and not individual) level.

#### Type of participants

‘Participants’ refers to all community members who may freely access community gardens. Participants were not limited to any particular subgroup of the community or by any characteristic (e.g. age, gender).

#### Type of intervention (for RCTs) or exposure contrast (for observational)

Community gardens were conceptualized as publicly accessible spaces that are used to grow vegetables and fruit. This definition included council median strips or verges that are made accessible and permissible for food production by the public, but excluded incidental use of verges by individuals for growing vegetables or fruit for personal purposes. The definition excluded production of crops for profit and animal-based food production, such as using community spaces for animals that produce milk and eggs, or for collection of honey. It also excluded fruiting trees on government properties (e.g. botanical gardens) or gathering native and non-native foods from national parks. The motivation for developing such gardens was not considered; whether they were designed for example, for food production in response to food insecurity issues in that area, or to create social and community connection. The key concept of the definition of community gardens was that they reflected *public access* to spaces; therefore studies that did not involve free access to the public were excluded (e.g. gardens in schools, hospitals or jails that are not freely accessible to the public).

#### Type of comparator

We adopted the counterfactual approach to understanding the effects of community gardens. For RCTs, the comparator was community members who did not receive the intervention (community gardens) and for observational studies, the comparator was non-exposed controls, or a pre-exposure group for pre-/post- designs.

#### Types of outcome measures

Outcomes were categorized as:

1. Food consumption, with particular attention to vegetable and fruit intake
2. Health outcomes, with particular attention to physical activity
3. Psychosocial measures, such as (but not limited to) social isolation, mental health and wellbeing
4. Community sentiment, such as (but not limited to) social cohesiveness

Characteristics community garden users and differences on effects of community gardens according to location (urban, regional, remote) or socioeconomic position were also explored.

### Screening

The titles and abstracts of all identified articles were examined using Rayyan software (a software program used to collate and screen papers for systematic reviews). The authors conducted the screening process and each title/abstract was viewed by two authors. Only articles that were irrelevant were excluded at this stage. The full text of the article was retrieved if either of the authors indicated that the title/abstract was eligible or unclear.

### Data extraction, management and synthesis

The full text of each article was reviewed and data were extracted systematically. For RCTs, study quality was evaluated using the Cochrane Risk of Bias tool [14] and the quality of non-randomized studies was assessed using the ROBINS-I tool [15]. A narrative meta-synthesis was undertaken because a meta-analysis was not possible due to differences in study designs and outcomes.

### Changes to the protocol

After commencing the searches it became apparent there were more systematic reviews on this topic than anticipated. A post hoc decision was made to include a table summarizing the main findings of each systematic review to collate the full body of literature. No assessment of the quality of each systematic review was undertaken since individual publications were being judged for quality as part of the current review.

## Results

**Fig. 1:**
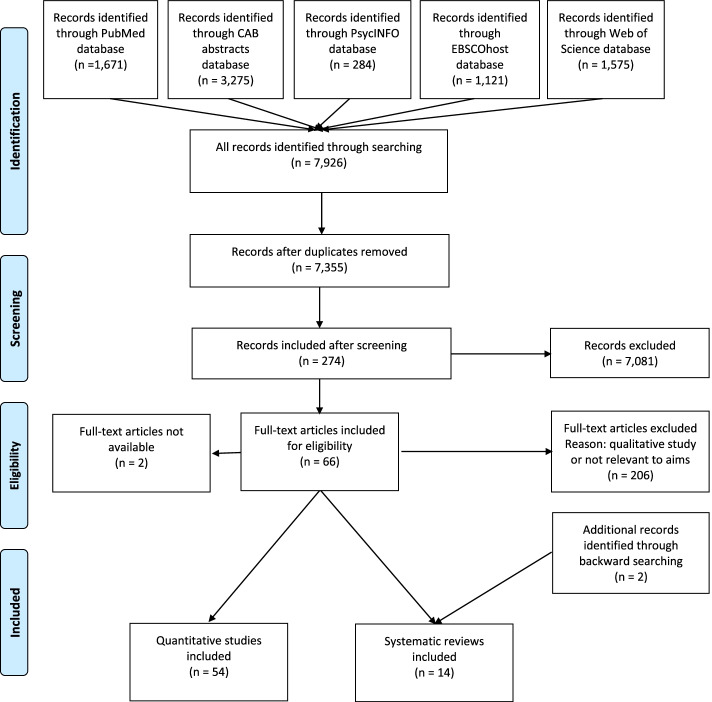
Flow of studies through the systematic review process

The search strategy captured 7,355 articles for screening after duplicates were removed. There were 66 papers judged as eligible for inclusion, but the full text was unable to be obtained for two papers. The flow of studies through the systematic review process is shown in Fig. 1. At least two authors extracted data from 12% of articles. Any discrepancies in data extraction or quality ratings were resolved by discussion at meetings involving all authors.

### Systematic reviews

The searches identified 14 systematic reviews in related topic areas. Table 1 shows the number of studies included in each review, as well as their aims and conclusions. These systematic reviews extended to areas beyond the scope of the current review (e.g. peri-urban agriculture), making only some components of these reviews directly relevant to our research aims. There were 10 to 196 articles included in these reviews. Nutrition and food security were the most commonly studied outcome (9/13 (69%)). The earliest systematic reviews indicated that various forms of community gardening had potential to improve fruit and vegetable intake and food security [16] although this view was not uniform with some suggesting gardens had little impact on food access [17]. Many systematic reviews agreed upon the poor quality of evidence [11, 12, 16, 18]. Recently, Spano et al. described that community gardening may benefit psychosocial wellbeing and this effect was more pronounced among individualist societies compared with collectivist societies [19].

**Table 1:** Summary of systematic reviews

Reference	Number of (*k*) studies included	Aims of the review	Conclusions of the review
Artmann et al. 2018 [20]	*k* = 196	To consider urban development (peri-) urban agriculture (UPA) as a nature-based solution for societal challenges. This systematic literature review investigates UPA in the global north and its impacts on societal challenges and co-benefits. Based on findings, it aims to develop an integrative assessment framework for evaluating the implementation of UPA	The value of UPA is its multifunctional nature; it contributes to food security, climate change, biodiversity and ecosystem services, sustainable agriculture, resource efficiency, urban regeneration, land management, public health, social cohesion, and economic growth. UPA can exist in many forms, such as public community gardening or semi-public allotments. When successfully managed, UPA can help urban residents reconnect with nature and reclaim public spaces
Audate et al. 2019 [11]	*K* = 101	The aim of this study was to explore the impacts of urban agriculture (UA) on the determinants of health and identify knowledge gaps for future UA studies by conducting a scoping review of peer-reviewed literature	More peer-reviewed studies are needed in areas where UA is practiced such as Latin America and Caribbean. The inconsistency and the lack of strong quality in the methodology of the included studies are proof that more rigorous studies are also needed in future research. Nevertheless, the substantial existing evidence from this review corroborate that UA can influence different determinants of health such as food security, social capital, health and well-being in a variety of contexts
Garcia et al. 2018 [9]	*k* = 24	To investigate how urban gardens impact healthy food practices, healthy food access, and healthy food beliefs, knowledge and attitudes	Participation in urban gardens was associated with positive outcomes on practices of adequate and healthy food intake and food perceptions. Findings suggest that community interventions may yield changes in knowledge and attitude by activating willingness for healthier food practices
Iacovou et al. 2013 [21]	*k* = 10	To investigate whether community kitchens can provide positive social and nutritional outcomes to participants and their families	Findings suggest that community kitchens can be an effective way of enhancing people’s cooking abilities, social networks, and nutritional intake. Community kitchens might also be able to improve budgeting skills of participants and alleviate concerns on food insecurity. However, there is a need for more rigorous qualitative and quantitative studies to effectively assess the issue
Kondo et al. 2018 [8]	*k* = 43	To evaluate stress responses to deliberate exposure to outdoor environments in the forms of nature viewing, outdoor walks, outdoor exercise, and gardening	Findings show that spending time outdoors, especially in green space, reduces the experience of stress and thereby enhances a person’s health
Kunpeuk et al. 2020 [12]	*k* = 19	To explore the relationships between community gardening, nutrition and physical health in adults	Findings suggested that community gardens produced significantly positive effects in fruit and vegetable consumption. For physical health outcomes, only some of the reviewed studies showed positive results. However, evidence was found that community gardening has a positive effect on BMI reduction. The study suggests the need for more research on the causal relationship between gardening and health outcomes if community gardens are to be integrated into health promoting policies at the population level
McCormack et al. 2010 [10]	*k* = 16	To provide an evaluation of the literature available on farmers’ markets and community garden programs and their potential to increase fruit and vegetable intake	Farmers’ markets and community gardens can increase access to fruit and vegetables, particularly in low-income areas where healthy foods are less accessible. By increasing access to fresh fruits and vegetables, diets can be improved. However, more research is required on the specific health benefits of these interventions
Optiz et al. 2016 [22]	*k* = 168	To provide an evaluation of the literature available on urban and peri-urban agriculture in the Global North, understanding their commonalities and differences as well as their influence on urban food security	Both urban and peri-urban agriculture are valuable to urban food planning but the two differ in outcomes. Urban agriculture meets the food needs of households whereas peri-urban agriculture can provide higher quantities of food to a larger population
Poulsen et al. 2015 [23]	*k* = 35	To assess the relationship between urban agriculture and food security in low income countries	Although participation in urban agriculture does not fully alleviate the pressure of food security, it can help women’s contribution to household food availability, and provide economic and social advancement. If agricultural policies support the incorporation of urban agriculture (including the integration of gender) then urban agricultural practice can become more effective. More research is required in settings where supportive policies have been enacted
Robinson-O’Brien et al. 2009 [16]	*k* = 11	To investigate the impact that garden-based youth nutrition programs have on nutrition-related outcomes	Findings from this review suggest that garden-based nutrition intervention programs may have the potential to increase fruit and vegetable intake among youth and increase willingness to try fruits and vegetables among younger children. However, there is a need for well-designed, evidenced-based, peer-reviewed studies to determine program effectiveness and impact
Schram-Bijkerk et al. 2018 [24]	*k* = 20	To assess the health effects of urban gardening through use of a framework	The study develops a set of indicators that assess ecosystem services and health impacts of urban gardens and can be useful in decision-making processes in urban management. The study suggests that urban gardens may improve the health of the community by overcoming the societal challenges of urbanization, health and well-being in aging populations and climate adaptation. Additionally, urban gardens form social-networks and can contribute to the cohesiveness of a community, promoting health and well-being
Smith et al. 2013 [17]	*k* = 29	To explore whether community gardens can increase healthy food accessibility in Metropolitan Atlanta communities	The study suggests that community gardens had a minimal impact on food access in urban communities. However, food policy advocacy and supermarket tax incentives were identified as effective ways to promote healthy community development
Spano et al. 2020 [19]	*k* = 7	To examine the effect of community gardening on outcomes related to psychosocial wellbeing	Nevertheless, an effect of publication bias and study heterogeneity has been detected. Despite the presence of a large number of qualitative studies on the effect of horticulture/gardening on psychosocial well-being, quantitative studies are lacking. There is a strong need to advance into further high-quality studies on this research topic given that gardening has promising applied implications for human health, the community, and sustainable city management
Warren et al. 2015 [18]	*k* = 13	To explore the association between urban agriculture and food security, dietary diversity, and nutritional status. Also, to examine whether urban agriculture is an effective solution to urban food insecurity	Causation could not be assigned due to the low quality or the study designs. Before urban agriculture can be recommended as a solution to urban food security challenges, more research needs to be conducted on the topic

### Diet and food-related outcomes

The 23 studies that reported diet and food-related outcomes are summarized in Table 2. Of these, the majority were conducted in the Unites States (16/23 (70%)), with two studies from France and one each from Canada, Japan, the Netherlands, South Africa and the United Kingdom. Most were cross-sectional surveys (15/23 (65%)), four were pre-/post reports of feasibility/pilot studies (4/23 (17%)), one quasi-experimental study, one longitudinal cohort study, and two pilot RCTs. Sample sizes varied from 20 [25] to 1000 [26].

**Table 2:** Studies addressing diet and food-related outcomes included in this review

First author, year	Country or setting	Study design	Sample characteristics (inclusion criteria, number, age and sex)	Aims	Sampling methods	Intervention / Community garden program	Data collection Analysis (including adjustments)	Outcomes	Results
Alaimo et al. 2008 [27]	Flint, MI, USA Rural and urban regions	Cross-sectional survey	766 adults Non-institutionalized Genesee County residents aged ≥ 18 yrs *n* = 845 Flint residents interviewed = 15% response rate Household participation in CG *n* = 116 vs not, *n* = 650 Mean (SD) Age: 46.4 (1.9) vs. 43.4 (0.8) yrs Male: 49.9 (5.4)% vs. 47.8 (2.2)% Female: 50.1 (5.4)% vs. 52.2 (2.2)% African American: 61.5 (5.3) vs. 46.6 (1.7) White: 26.4 (4.7)% vs. 43.8 (1.9)% Other: 12.1 (4.4)% vs. 9.6 (1.4)%	To determine the association between household participation in a CG and F&V consumption among urban adults	Survey administered by telephone biennially Quota sampling strategy	None	F&V intake (Behavioral Risk Factor Surveillance System); Household participation in a CG Generalized linear models and logistic regression models, controlling for demographic, neighborhood participation, and health variables	Fruit and vegetable consumption relative to national recommendations	F&V consumption: 4.4 (0.3) vs. 3.3 (0.1) times per day Consumed F&V ≥ 5x/d (national recommendations): 32.4 (4.9)% vs. 17.8 (1.7)% Respondents with a household member who participated in CG consumed F&V 1.4x/d more, and were 3.5 × more likely to consume F&V ≥ 5x/d vs. those without a gardening household member
Algert et al. 2016 [28]	USA, California, San Jose	Cross-sectional survey	Two groups: *Characteristics* Community gardeners: *n* = 85) 84% female Age 49 (± 13) yrs Home gardeners *n* = 50 50% female Age 58 (± 12) yrs	To compare whether the two groups of gardeners (community and home) increased their vegetable intake while gardening	1) CG: Face-to-face recruitment at 4 separate allotments 2) La Mesa Verde (LMV): Recruited through existing home gardening project for low-income families Response rate not reported	No INT; 2 CG programs	T-tests and Chi-square test comparing veg intake btw home and community gardeners No adjustments	Vegetable intake (EFNEP food behavior checklist)	Results of statistical analyses not reported Intake of vegetables similar between groups (1.9 and 2.0 cups/day for home and community gardeners respectively), increased when majority of participants reported eating from the garden (4.0 cups/day)
Barnidge et al. 2013 [26]	USA, rural Missouri, 7 counties	Cross-sectional surveys	Two groups: Community gardeners: *Characteristics* *n* = 141 Male: 28.4%e Age: 72.3% > 45y Ethnicity: 54.6% non-Hispanic white Education: 53.2% ≥ high school equivalency CG exposure: 63.8% ≥ weekly Phone survey: *Characteristics* *n* = 1000 Male: 26.6% Age (mean): 59.7y Ethnicity: 88.0% non-Hispanic white Education: 43.9% ≥ high school equivalency CG participation: 95.4% do not participate in CGs *Inclusion criteria* None reported	To examine relationship between CG participation and F&V consumption	Community gardeners: Intercept survey with known community gardeners Phone survey: Random digit dial sample from 16,000 landlines in 5 towns with community gardens in a 5 mile radius	N/A	Chi-square tests, no adjustment Multivariate logistic regression models, adjusted for sex, race, age, education, social cohesion, sense of belonging and food environment	F&V consumption, eating fresher food, eating less fast food,	Comparisons between gardening frequency (< once/ wk vs once/wk or more) and outcomes Frequent gardeners eat more F&V (χ^2^ = 7.78; *p* = 0.088), eat fresher food (χ^2^ = 15.38) and eat less fast food (χ^2^ = 5.19) CG participation associated with: Increased odds of meeting F&V recs in fully adjusted model (OR = 2.76, 95%CIs = 1.35–5.65)
Barnidge et al. 2015 [29]	USA, rural MO, 2 counties	Quasi-experimental study	Total *n* = 794 Inclusion criteria: African American, ≥ 18 y Residing in COM. or INT county *Characteristics* INT: *n* = 397 Female: 62.7–63.2% (baseline and mid INT, respectively) COM: *n* = 397 female: 65.0–71.3% (baseline and mid INT, respectively) Age (mean): 38.8–41.7y	To examine effect of INT on BP, self-reported BMI, F&V consumption (Mid-INT results)	Cross-sectional surveys at each time point in INT and COM county Recruited from “places frequented by African American adults (e.g. community organization or church)”, fliers posted	MOTMGC (Men on the Move Growing Communities) – existing CG, nutrition education activities; access to healthy food through CG (participants did not do gardening themselves); 3 production gardens	Self-administered survey Logistic regression models: changes over time between counties in prevalence of hypertension and BMI; models adjusted for age, education, employment and income	F&V consumption	Increased odds of eating 5 + servings of F&V daily for high (OR: 3.06; 95%CIs: 1.90–4.95) and medium nutrition education participation (OR: 1.98; 95%CIs: 1.42–2.76), compared to no participation Increased consumption of F&V for those receiving F&V from CG compared to not (OR: 1.95; 95%CIs 1.20, 3.15) in fully adjusted models Strongest effect on F&V consumption from high participation AND receiving F&V from CG, compared to others (OR: 2.18; 95%CIs: 1.24, 3.81)
Brown et al. 2020 [25]	USA, MT, Native American community	RCT (1) Group-based Community gardening program (2) control (no gardening program)	Native Americans with prediabetes or diabetes CON: *n* = 12 INT: *n* = 8 Age: *N* = 15 were 45–64 y; *n* = 5 were 25–44 y Male n(%): 4 (25%)	To determine feasibility of a group gardening program and potential for collecting health outcomes	Convenience sample of participants expressing an interest in the gardening study at a diabetes clinic	Raised beds for gardening chosen for proximity to college and health centre. Plus 10 × 90-min structured sessions with hands-on gardening and food preparation activities Outcomes measured at 7 months after baseline	Outcomes were reported as medians and ranges. Change from baseline was compared between the groups using Wilcoxon rank sum tests. No adjustments Missing information on some outcomes	Diet: Motivation to eat fruits and vegetables	Change from baseline Motivation to eat F&V (median [range]) INT (*n* = 6): 0 [-1.0, 5.0] CON (*n* = 11): 0[-2.0, 3.0] P = 0.838
Carney et al. 2012 [30]	USA, OR, Columbia River Gorge (rural farm community)	Pre-/post (no control group)	*n* = 38 families at baseline (*n* = 163 individuals) *Characteristics* Age (mean): 44 y (21–78) Average yrs living in US: 20 (4–44) *Inclusion criteria* None reported	To study the impact of a CG program on vegetable intake (also food security and family relationships) of migrant seasonal farmworker (rural) families	All families volunteered for the program No recruitment methods reported	Community meetings held at start of growing season to provide materials (e.g. seeds) and information on gardening techniques, and concerns about exposure to pesticides	Pre-post survey was interviewer- administered to nominated family member (phone or face to face) Instrument examined frequency of eating vegetables. No validity, reliability or source reported Wilcoxon signed rank test examined pre-post responses. No adjustments reported	Frequency of adult and child vegetable intake	Frequency of adult veg intake of “several times a day” increased from 18.2 to 84.8% (*p* < 0.001) Frequency of child veg intake “several times a day” increased from 24.0 to 64.0%
Castro et al. 2013 [31]	USA, NC, Carrboro	Pre-/post (no control group)	*n* = 60 families *n* = 120 children *Characteristics* Male: 49% boys Ethnicity: 59% Latino Age (mean): 6.0 (± 3.4)y *Inclusion criteria* Families living in the community (Carrboro); had ≤ 1 child ≥ 6 y	1. To help children achieve or maintain a healthy BMI 2. To increase children’s access to fruit and vegetables, particularly at home 3. To increase the daily number of servings of fruit and vegetable children consumed	Families recruited through outreach activities at schools and other local service providers	Growing Healthy Kids (GHK)—3 yr program consisting of: 1) weekly gardening sessions; 2) cooking and nutrition workshops for parents and children; 3) social activities and events	Surveys administered at baseline and at end of each year Change in proportion of positive outcomes pre and post for: - Availability of F&V - F&V consumption	F&V intake Availability of F&V	Fruit consumption: Increased by 28%/d (2 extra serves/week; t = 4.31; df = 47; *p* < 0.001) Vegetable consumption: Increased by 33%/day (4.9 extra serves/week; t = 3.17; df = 45; *p* < 0.001) Fruit availability: Increased by 146%; average absolute change = 2.55 (SD = 1.41) (t = 12.53; df = 47; *p* < 001) Veg availability: increased by 123%; av. absolute change = 4.3 (SD = 1.82) (t = 16.37; df = 47; *p* < 0.001)
De Marco et al. 2016 [32]	USA, NC, Rural low resource county	Pre/post study	n- = 40 *Characteristics* Rural African American youth *n* = 17 Rural African American adults *n* = 23 *Inclusion criteria* Open to adults and youth ≥ 10 y	To test the feasibility of a church garden program to impact health outcomes in rural African American youth and adults	Assistant pastor recruited known church and community members	Workshops 2 h/wk; hands-on gardening and nutrition education	Paired P-tests examined within group differences (pre-post) for adults and youth separately	Food-related knowledge; attitudes; perceptions; behaviors Weight, BMI, BP	Youth (*n* = 14) F&V knowledge increased (12.9 to 14.5, *p* = 0.08) Daily Vegetable intake increased: (2.25 to 2.5 serves, *p* = 0.08) Adults (*n* = 20) F&V knowledge: 20.3 to 21.1 Daily F&V intake*:* 2.3 servings to 2.5 servings
Hartwig and Mason 2016 [33]	USA, MN, Twin Cities	Cross-sectional surveys	*n* = 97 *Characteristics* Female: 65% Good/fluent English: 18% Age (mean): 39y (16–80 y) Ethnicity: 67% Karen (Burmese) *Inclusion criteria* None stated	To evaluate church community gardens serving refugee and immigrant populations, reporting primary health and social benefits	8 Gardens purposively sampled based on: - 2 yrs participation - # gardeners - primary language of gardeners (Karen & Nepali) All gardeners at 8 gardens invited at beginning and end of season (two samples) Response rate = 44–45%	8 church gardens serving refugees and immigrants	Measured early and late season harvest (Jul-Sept) Change in mean/% early and late season No adjustments	F&V intake Food security	% reporting F&V intake everyday Increased from 64 to 78% 4% reported food security issues (but 86% on food subsidy programs)
Heilmayr and Friedman 2020 [34]	USA, CA	RCT with 5 INT groups: (1) Community gardening (2) moderate indoor exercise (3) Exposure to nature (4) Social club (watching films) (5) Indoor container gardening	University students Baseline data reported in combination (not by group allocation) *Characteristics* Age: 20.6 ± 3.3y Male: 31.2% (1) *n* = 21 (2) *n* = 21 (3) *n* = 23 (4) *n* = 22 (5) *n* = 23	To compare community gardening with four theoretically driven comparison groups to understand possible causal mechanisms around how community gardens have improved outcomes	Convenience sample recruited via flyers, emails and the Psychology Subject Pool	4 week INT; assigned an activity for 2–3 h/wk	Data were analysed by ANOVA with pre-/post-test values to assess how groups changed from baseline and a group by time interaction	Items from a Food Frequency Questionnaire to generate an overall score of produce consumption (items NR)	Produce consumption (post-test only; mean ± SD) (1) 4.6 ± 1.5 (2) 4.8 ± 1.5 (3) 4.9 ± 1.7 (4) 5.3 ± 1.8 (5) 5.0 ± 1.7
Hopkins and Holben 2018 [35]	USA, OH, Athens (rural Appalachia)	Cross-sectional survey	*n* = 50 *Characteristics* Ethnicity: 81.6% white Female: 67.4% Education: 46.9% college educated *Inclusion criteria* CG plot in Athens	To examine relationships among food security, produce intake and behaviours, health and social capital among community gardeners	All community gardeners (*n* = 120) in Athens invited, Response rate = 42%	No INT	Survey distributed via email Descriptive statistics, no adjustment	F&V intake Food security	46% eat more F&V due to CG 79.1% have high food security Food insecure gardeners ate more F&V due to CG compared to secure gardeners (tau = 0.285, *p* = 0.03)
Kim et al. 2017 [36]	UK, London	Cross-sectional survey	*n* = 48 *Characteristics* Female: 66.7% Length of gardening: 37.5% for ≥ 5 yrs	To examine relationship btw CGs and daily food consumption, in relation to carbon footprint	95 CGs and food growing organizations in London contacted to distribute survey via email	No INT Individuals participating in CGs	Descriptive statistics Sample divided into 3 groups by yrs of participation in CGs	Meat consumption; dining out; convenience food consumption; food self-sufficiency; growing food outside CG	27.7% ate meat never or < once/week; most ate meat 1-3x/wk (31.9%) or 4-6x/wk (25.5%) 68.1% ate out < 1x/wk ~ 94% ate convenience foods < 3x/wk 58.7% grew food outside CG; highest among longest gardeners (61.11%) - 57% said food from CG was helpful or very helpful to decrease food purchasing; highest among longest gardeners (66.67%)
Litt et al. 2011 [37]	USA, CO	Cross sectional survey	*n* = 436 *Characteristics* Ethnicity: 57% White Female: 68% Education: 56% College educated *Inclusion criteria* English- or Spanish-speaking adults aged ≥ 18 y	Provide insights into (1) social and psychological factors that shape F&V consumption in an urban setting and (2) community-based healthy eating strategies that address those factors	Multi-frame sampling design Area-based sample of general population and a list-based census of community gardeners. All households located within 1 mile of CG Response rate = 59%	No INT	Multilevel analytic models; adjustments included education, physical activity, BMI, and self-rated health	F&V intake, physical activity, BMI, SEP, and dimensions of health Self- developed measure of F&V intake (6 items) asking about frequency of intake, including fruit juice	Mean health variables - F&V consumption: 4.4x/day -17 h/wk PA - BMI 26.2 kg/m^2^ 9% community gardeners Comparisons to other gardeners - Community gardeners consumed F&V 5.7x/d vs. home gardeners (4.6x/d) and non-gardeners (3.9x/d) - 56% of community gardeners consumed F&V ≥ 5x/d, vs. 37% of home gardeners and 25% of non-gardeners
Litt et al. 2015 [38]	USA, CO, Denver	Cross-sectional survey	*n* = 469 *Characteristics* Age (mean): 46.1y (± 15.9) Female: 67.4% Education: 57.4% college educated Identified as gardeners: 59.3% *Inclusion criteria* English or Spanish speaking, ≥ 18yrs	To examine the direct and indirect pathways by which gardening influenced self-rated health	Multi-frame sampling design Area-based sample and list-based census of community gardeners Response rate = 59%	No INT Individuals participating in CGs compared with non-gardeners	Surveys interviewer administered Path analysis comparing community gardeners with non-gardeners, Analyses controlled for age, education, yrs in neighborhood, observed incivilities	F&V intake Self-rated health	Data fit model adequately, accounting for 22% variance in self-rated health and 4% in F&V intake Gardening predicted F&V intake (β = 0.21, *p* < 0.001)
Machida 2019 [39]	Japan	Cross-sectional survey	*Characteristics* (1) Community gardeners *n* = 129 Male n (%): 87(67%) Age (mean): 64.1y ± 2.6 (2) Home gardeners *n* = 371 Male n (%): 280(76%) Age (mean): 63.9y ± 2.7 (3) Non-gardeners *n* = 500 Male n (%): 327 (65%) Age (mean): 63.3y ± 2.5 *Inclusion criteria* Aged 60–69 *Exclusion criteria* Professional farmer	To study the relationship between community or home gardening and health status or a healthy lifestyle	The web-based survey was conducted by a marketing company with 4.2 million people registered across all 47 prefectures in Japan	No INT	Odds Ratios adjusted for sex, age, family structure and employment status (not described)	Breakfast (everyday versus not every day) Vegetable intake (enough + moderate versus not enough + shortage) Frequency of eating balanced meals with grain, fish and meat, vegetables (eat every day versus not every day)	(Ref: non-gardeners) Eats breakfast every day (OR (95%CI) (1) Community gardeners: 1.94 (1.10, 3.43) (2) Home gardeners 1.21(0.59, 2.48) Eats enough vegetables (OR (95%CI) (1) Community gardeners 2.29 (1.67, 3.14) (2) Home gardeners 1.83(1.19, 2.85) Eats balanced meals everyday (OR (95%CI) (1) Community gardeners: 1.80 (1.33, 2.44) (2) Home gardeners 1.48 (0.97, 2.27)
Mangadu et al. 2017 [40]	USA, NM, US-Mexico border areas	Cross-sectional survey	Two community gardens accessible by the public. (CG1, CG2) CG1 (*n* = 16) CG2 (*n* = 9) % Male NR Age NR CG2 is a local government project comprising a neighborhood community garden and a garden on a juvenile probation campus. Where possible, data from the probation campus are not extracted	To identify the best practices in implementing and increasing the potential or sustainability of CGs	NR	NR	Descriptive statistics No adjustment	Nutrition data from Food Security Coalition’s Community Gardener/Farm-to-School survey but adapted (unclear how) to each community project. For nutrition (-items, yes/no responses)	Do you consume more F&V as a result of CG participation: CG1: Yes, *n* = 15/16 (94%) CG2: yes, NR
Martin et al. 2017 [41]	France, Marseille, socioeconomically disadvantaged northern districts	Cross-sectional survey	Five CGs close to social housing *Characteristics* Gardeners *n* = 21 Male: 0% Age (mean SD): 52y ± 12 Non-gardeners: *n* = 65 Male: 0% (all males excluded from analysis) Age: NR	To test whether, in poor neighborhoods, community gardeners have a greater supply of fruits & vegetables than non-gardeners	223 active gardeners invited. Non-gardeners were residents of the same neighborhood who participated in a nutrition education program	Arrays of plots that are cultivated individually. Most were growing Mediterranean fruits and vegetables	Generalized linear model with adjustment for age and number of children in the household	Total F&V intake measured as g/person/d The intake combines purchased (and harvested for gardeners)	Total F&V (g) purchases per person per day (mean ± SD) Gardeners 370 ± 283 Non-gardeners 211 ± 155
Roncarolo et al. 2015 [42]	Canada, Montreal,	Cross-sectional survey	Participants sampled from 16 traditional (e.g. food banks, *n* = 711) or 6 alternative (e.g. community gardens) venues (*n* = 113) *Characteristics* Female: 55% Age: 52% aged 30–49 yrs	To compare outcomes between users of traditional versus alternative organizations	Sampled from food security organizations with ≥ 50 new members (traditional) or ≥ 30 new members (alternative)	Not precisely described but indicated as being organizations (gardens) that nurture solidarity, and have goals of reducing social inequalities	Multilevel logistic regression to account for clustering by study site. Adjusted for sex, country of birth, marital status, employment, education, income and number of people in the household	Food security using the Canadian Community Health Survey (18 yes/no items)	Food security Ref = Secure Moderately insecure: OR_adjusted_ = 0.16 (0.08, 0.35) Severely insecure OR_adjusted_ = 0.09 (0.04, 0.20)
Schmidt et al. 1995 [43]	South Africa, Kudumane district	Cross-sectional survey	Poor rural area. Children whose parents participated in a communal vegetable garden (*n* = 18, INT) or not (*n* = 18; CON) *Characteristics* Male: % NR Age: 6–13 yrs	To investigate whether people who grow their own vegetables eat more vegetables and have better nutritional status than those who don’t	NR	INT: Trench gardens, 6 per household CON: purchased vegetables from shops	24-h recalls, fasting blood sampling for nutrient status No adjustments	24-h recalls: vegetable intake, energy, protein fat and fibre Blood sampling for vitamin A, β carotene, vitamin E, vitamin B6	Frequency of vegetable consumption: data NR *Experimental vs. Control (mean* ± *SD)* Energy 718 ± 413 kcal vs. 834 ± 472 kcal Protein 25.6 ± 22.2 g vs 26.6 ± 17.6 g Fat 2.8 ± 14.9 g vs. 9.7 ± 17.6 g Fibre 9.1 ± 5.3 g vs. 9.6 ± 6.9 g Vitamin A 1.23 ± 0.48 µmol/L vs. 1.21 ± 0.56 µmol/L Carotene 0.07 ± 0.06 mg/mL vs. 0.09 ± 0.15 mg/mL Vitamin E 8.75 ± 4.06 µmol/L vs. 6.51 ± 2.89 µmol/L Vitamin B6 21.2 ± 5.1 ng/mL vs. 20.2 ± 2.0 ng/mL
Spees et al. 2016 [44]	USA, OH, Columbus; Adult cancer survivors	Pre-/Post	*n* = 22 *Characteristics* Age (mean) 62y; Age (mean) initial cancer diagnosis: 59y *Inclusion criteria* Adults ≥ 18 yrs, English speaker, access to Internet, basic computer skills, and cancer survivors who had completed active cancer treatment (chemotherapy, radiotherapy, and/or surgery) within previous 24 months	To determine the feasibility, acceptability, and preliminary efficacy of a multifaceted, evidence-based intervention for cancer survivors transitioning out of active treatment and orchestrated around a season of herb, fruit, and vegetable harvesting in an urban garden	Adult cancer survivors recruited from the James Cancer Hospital and Solove Research Institute	4-month multifaceted INT focusing on cancer survivor–specific nutrition, PA, and behavioral modification delivered within a garden setting Garden was 2.5-acre plot with herbs, F&V	Effect of INT on outcomes were conducted by comparing the pre-study and post-study scores	Medical, dietary (26-item Dietary Screener Questionnaire) Objective anthropometric and fasting clinical biomarkers	Post INT: Increased F&V consumption (~ 3.5 cups to 4.2 cups) Decreased added sugars consumption (~ 1tsp down to 0.9 tsp) Decreased intake red and processed meat (0.3 units down to 0.2 units)
Spliethoff et al. 2016 [45]	USA, New York City (NYC)	Cross-sectional	NYC community gardeners *Characteristics* *n* = 46 (information on a total of 93 adults and 13 children in their households) Age: NR *Inclusion criteria* NR	To assess vegetable consumption rates and time spent in the garden in NYC community gardeners	Mailing to contact gardeners at 76 NYC CGs from which soil had been sampled (separate aim) and to volunteers at NYC gardening workshops	No INT	Median and 95th percentile consumption rates for crops (fruiting, leafy, root, and herb) for gardeners (*n* = 46), and adult (18 + yrs; *n* = 47) and child (< 18 yrs; *n* = 13) household members Lognormal distributions to consumption rates for each crop type (consumers only)	Description of crop grown in past 12 months and estimate crop harvested during that time; estimate fractions of harvest consumed/not consumed by themselves plus by household; age, body weight; servings of F&V	89% of gardeners and child household members, and nearly all adult household members ate at least some vegetables from their CG Community gardeners (*n* = 46) Total vegetable intake (mean ± SD): 1308 mg/kg day, made up of fruit (353 ± 4.8), leafy (220 ± 3.2), root (85 ± 3.1), herb (39 ± 3.4) vs. nationally representative consumption rates for home-produced vegetables (mea*n* = 2020 mg/kg day) Age and body weight NR
Tharrey et al. 2020 [46]	France, Montpellier	Longitudinal (1 yr) cohort study	*Characteristics* (1) Community gardeners (*n* = 66) Male n(%): 16(24.2) Age (y): 44.0 ± 14.0 (2) Non-gardeners (*n* = 66) Male n(%): 16(24.2) Age (y): 44.9 ± 13.7 *Inclusion criteria* Starting gardening in a community garden; residents of Montpelier; ability to read French	To assess the impact or urban community garden participation the adoption of sustainable lifestyles	Gardeners recruited when new to the gardening community Non-gardeners recruited via volunteers for a population-based survey on food supply behaviors Matched on age, sex, household income and household composition	No INT	Analyzed with mixed-effects models with group by time interaction Adjustments for education, BMI, meals consumed outside the home, social desirability where appropriate	Grams of F&V consumed(g/pp/d) 20 essential nutrients (Mean Adequacy Ratio (MAR)) Sodium, free sugars and saturated fatty acids (Mean excess Ratio (MER)) Household purchasing index (HPI)	F&V data at 1 year follow up (g/pp/d; mean ± SD) (1) 400 ± 231 (2) 446 ± 305 NS MAR at 1 year follow-up (percent adequacy/ 2000 kcal; mean ± SD) (1) 75.8 ± 8.1 (2) 76.9 ± 6.5 NS MER at 1 year follow-up (percent excess/2000 kcal; mean ± SD) (1) 96.1 ± 23.4 (2) 98.8 ± 29.7 NS HPI at 1 year follow-up (mean ± SD) (1) 9.0 ± 2.1 (2) 9.1 ± 1.9 NS
Veen et al. 2016 [47]	The Netherlands	Cross-sectional	6 gardens *n* = 237 *Inclusion criteria* NR	To investigate the extent to which CGs influence the enhancement of social cohesion	Gardens selected to ensure homo- and heterogeneity in neighborhood, plot type and harvest consumption type Recruitment via newsletter and letter to CGs	No INT	F-statistic, generalized linear models, chi-square No adjustments	Motivation for gardening (vegetables; social atmosphere, gardening hobby)	Higher motivation for vegetables associated with higher vegetable consumption (*p* < 0.001)

*Abbreviations BMI* Body mass index, *CG* Community garden, *COM* Comparison group, *CON* Control group, *F&V* Fruit and vegetable, *INT* Intervention group, *NR* Not reported, *OR* Odds ratio, *PA* Physical activity, *RCT* Randomized controlled trial, *SD* Standard deviation, *SE* Standard error, *SEP* Socioeconomic position

Studies that compared community gardeners with non-gardeners generally reported higher fruit and vegetable consumption by gardeners [26, 27, 30, 31] or with higher frequency of gardening [26]. However comparisons between community gardeners and home gardeners indicated that fruit and vegetable consumption did not differ [28]. Some community gardeners grew food outside of the community garden [36].

### Health outcomes

Table 3 summarizes the 16 studies reporting health-related outcomes (one of these studies was reported in two papers). Eleven (11/16 (69%)) of these studies were conducted in the United States, with two studies from Japan, 1 each from France, the Netherlands and the United Kingdom. Most were cross-sectional surveys (9/16 (56%)), in addition to 3 pre-/post- designs, one quasi-experimental, one longitudinal and two RCTs. Studies ranged in size from 13 participants at follow up [48] to 794 [29]. The diverse outcomes reported in these studies included weight-related outcomes such as BMI, overweight and obesity, self-reported outcomes such as health, physical activity, number of general practitioner visits and the number of chronic illnesses, and clinical measures of hypertension and blood glucose (HbA1_c_).

**Table 3:** Studies addressing health outcomes included in this review

First author, year	Country,setting	Study design	Sample characteristics (inclusion criteria, number, age and sex)	Aims	Sampling methods		Intervention/ Community garden program	Data collection Analysis (including adjustments)	Outcomes	Results
Algert et al. 2016 [28]	USA, CA, San Jose	Cross-sectional survey	Two groups: *Characteristics* Community gardeners: *n* = 85 Female: 84% Age (mean (± SD): 49 (± 13)y Home gardeners (HG) *n* = 50 Female: 50% Age (mean (± SD): 58 (± 12)y	To compare whether the two groups of gardeners (community and home) increased their vegetable intake while gardening	1) CG: Face-to-face recruitment at 4 separate allotments 2) La Mesa Verde (LMV): Recruited through existing home gardening project for low-income families Response rate not reported		Participants in 1) San Jose’s CG program which provides space to grow food, socialize and learn about gardening) 2) Local govt. funded (LMV; home gardening project) which provides raised beds, soil, seeds and plants; instruction on organic gardening workshops	T-tests and Chi-square test comparing home and community gardeners No adjustments	Self-reported health status (BRFSS) and BMI	Self-reported health Community gardeners: Excellent to very good 35% Good 48% Fair/poor 17% Home gardeners: Excellent to very good 45% Good 35% Fair/poor 20% BMI Community gardeners: 26.3 ± 5.3 Home gardeners: 28.5 ± 6.0
Barnidge et al. 2015 [29]	USA, rural Missouri, Dunklin (COM) and Pemiscot (INT) counties	Quasi-experimental study	Total *n* = 794 (397 COM; 397 INT) *Characteristics* INT group Female: 62.7–63.2% COM group: Female: 65.0–71.3% Age: 38.8–41.7y *Inclusion criteria* African American, ≥ 18 yrs, residing in COM or INT county	To examine effect of INT on BP, self-reported BMI, F&V consumption (Mid-intervention results)	Cross-sectional surveys at each time point in INT and COM county Recruited from “places frequented by African American adults (e.g. comm. org or church)”, fliers posted		MOTMGC (Men on the Move Growing Communities) – existing CG, nutrition education activities; access to healthy food through CG (participants did not do gardening themselves); 3 production gardens	Self-administered survey Logistic regression examined changes prevalence of hypertension and BMI between INT and COM counties; models age, education, employment and income incl. in models to calculate adjusted changes over time between counties	BP directly measured BMI from self-reported height and weight	Odds of hypertension: Decreased in INT county (OR: 0.52; 95%CIs: 0.38–0.71) but not in COM county (OR: 1.11; 95%CIs: 0.81–1.54) in fully adjusted models Odds of being overweight or obese: Declined in INT county (OR: 0.73; 95%CIs: 0.52–1.02) but not in COM county (OR: 1.30; 95%CIs: 0.89–1.91) in fully adjusted models
Brown et al. 2020 [25]	USA, Montana, Native American community	RCT (1) INT: Group-based CG program (2) CON: No gardening	Native Americans with prediabetes or diabetes *N* = 20 Age (y): 15/20 were 45–64 years, 5/25 25–44 years Male n (%): 4/20 (25%) CON *n* = 12 INT *n* = 8	Determine feasibility of a group gardening program and potential for collecting health outcomes	Convenience sample of participant expressing an interest in the gardening study at a diabetes clinic		Raised beds for gardening chosen for proximity to college and health centre. Plus 10 × 90-min structured sessions with hands-on gardening and food preparation activities Outcomes measured at 7 months after baseline	Outcomes were reported as medians and ranges. Change from baseline was compared between the groups using Wilcoxon rank sum tests. No adjustments Missing information on some outcomes	Weight, BMI, HbA1c, systolic and diastolic blood pressure (SBP and DBP)	BMI INT (*n* = 8): -0.69 [-1.9, 0.3] CON (*n* = 12): 0[-2.0, 3.0] P = 0.838 SBP INT (*n* = 7) -1.0 [-6.0, 16.0] CON (*n* = 12) -9.0 [-28, 24] P = 0.444 DBP INT (*n* = 7) -6.0 [-18.0, 12.0] CON (*n* = 12) -3.0 [-22, 10] P = 0.983 HbA1c INT (*n* = 8) -0.25 [-0.06, 0.9] CON (*n* = 12) -0.2 [-2.6, 5.6] P = 0.925
Castro et al. 2013 [31]	USA, NC, Carrboro	Pre-/post (no CON)	*Characteristics* 60 families participated *n* = 120 children Boys: 49% Ethnicity: 59% Latino/a Age (Mean (± SD): 6.0 (± 3.4)y *Inclusion criteria* Families living in the community (Carrboro); had ≤ 1 child 6 + y	1. To help children achieve or maintain a healthy BMI 2. to increase children’s access to fruit and vegetables, particularly at home 3. To increase the daily number of servings of F&V children consumed	Families recruited through outreach activities at schools and other local service providers		Growing Healthy Kids (GHK)—3 yr program consisting of: 1) weekly gardening sessions; 2) cooking and nutrition workshops for parents and children; 3) social activities and events meetings; newsletter; etc.)	Height and weight collected pre-and post-program (3y) Surveys administered at baseline and at end of each year. Survey was piloted with focus groups and previously been used with Latino families	Change in BMI	Changes in BMI 17% of obesity (*n* = 6) resolved 23% of overweight (*n* = 3) resolved 100% of healthy weight (*n* = 53) maintained healthy weight
De Marco et al. 2016 [32]	USA, NC, Rural, low resource county	Pre-/post design June 2010-May 2011 (11 months)	*Characteristics* Rural African American youth (*n* = 17) and adults (*n* = 23) *Inclusion criteria* Open to adults and youth ≥ 10 y	To test the feasibility of a church garden program to impact health outcomes in rural African American youth and adults		The assistant pastor recruited church members and community members known to him	Workshops 2 h/wk; hands-on gardening and nutrition education	BP, height, weight, BMI Assessed using paired t-tests	Weight, BMI, blood pressure	Youth (*n* = 14) Weight: 148.5 lb to 151.9 lb BMI percentile: 71.3 to 71.7 Systolic BP: 120.5 to 113.5 Diastolic BP: 74.6 to 73.3 Adults (*n* = 20) Weight: 204.7 lb to 202.2 lb BMI: 32.5 to 31.7 Systolic BP*:* 137.5 to 136.6 Diastolic BP: 84.3 to 83.8
Hawkins et al. 2011 [49]	UK; Cardiff, Wales	Cross-sectional study (1) Indoor exercise group (2) Walkers (3) Allotment gardeners (4) Home gardeners	*Characteristics* (1) *n* = 23 Age (y): 72.9 ± 6.9 Male: 3 (13%) (2) *n* = 25 Age (y): 62.4 ± 6.8 Male: 8 (32%) (3) *n* = 25 Age (y): 65.7 ± 9.1 Male: 17 (68%) (4) *n* = 21 Age (y): 69.5 ± 7.7 Male: 2 (10%) *Inclusion criteria* ≥ 50 y attending various local activity groups	Measure health status and perceived stress of allotment gardeners compared to other activity groups (indoor exercisers, walkers, home gardeners)	Recruited via leaflets, posters and visits to groups from researcher Response rate 87.8%		Compared leisure activity groups to members of allotment gardening group No intervention	Self-reported health using the SF-36v2; PA (MET (min/wk) and sitting time measured using the International Physical Activity Questionnaire); BMI, BP, forced vital capacity (FVC; a measure of lung function)	Physical activity, sitting, self-reported health, BMI, pulse pressure calculated from P_systolic_ – P_diastolic_), lung function	No group differences in health outcomes Self-reported physical health (median; IQR) (1) 48.3 (41.2–55.9) (2) 51.6 (43.9–54.1) (3) 53.5 (43.2–57.9) (4) 50.0 (45.3–56.2) PA (MET min/week; median (IQR)) (1) 3576 (2076–5760) (2) 3450 (2232–6985) (3) 5915 (2428–11,196) (4) 3282 (1724–5630) Sitting time (min/wk; mean ± SD) (1) 346 ± 210 (2) 356 ± 183 (3) 305 ± 139 (4) 371 ± 190 BMI (mean ± SD) (1) 26.2 ± 5.2 (2) 26.9 ± 4.3 (3) 25.5 ± 3.3 (4) 27.3 ± 2.2 Pulse pressure; mean ± SD) (1) 64.3 ± 15.4 (2) 54.6 ± 14.2 (3) 62.4 ± 16.3 (4) 63.7 ± 15.1 FVC (mean ± SD) (1) 94.8 ± 25.4 (2) 99.4 ± 34.2 (3) 104.9 ± 33.3 (4) 93.6 ± 21.9
Heilmayr and Friedman 2020 [34]	USA, CA	RCT with 5 INT groups: (1) Community gardening (2) moderate indoor exercise (3) Exposure to nature (4) Social club (watching films) (5) Indoor container gardening	University students *Characteristics* Baseline data reported in combination (not by group allocation) Age (y): 20.6 ± 3.3 Male: 31.2% (1) *n* = 21 (2) *n* = 21 (3) *n* = 23 (4) *n* = 22 (5) *n* = 23	To compare community gardening with four theoretically driven comparison groups to understand possible causal mechanisms around how community gardens have improved outcomes	Convenience sample recruited via flyers, emails and the Psychology Subject Pool		4 week INT; assigned an activity for 2–3 h/wk	Data were analysed by ANOVA with pre-/post-test values to assess how groups changed from baseline and a group by time interaction	Self-reported health; Sleepiness; PA Fatigue Short Form a (4-items, responses NR), Body mass index	Self-reported health (post-test only; mean ± SD) (1) 63.2 ± 18.8 (2) 63.9 ± 17.6 (3) 61.9 ± 17.9 (4) 61.0 ± 17.1 (5) 64.0 ± 16.2 Sleepiness (post-test only; mean ± SD) (1) 9.2 ± 4.1 (2) 7.7 ± 4.9 (3) 8.8 ± 5.5 (4) 9.3 ± 3.1 (5) 9.3 ± 4.0 PA (post-test only; mean ± SD) (1) 2.8 ± 1.2 (2) 3.1 ± 1.2 (3) 3.1 ± 1.2 (4) 3.1 ± 1.2 (5) 3.4 ± 1.6
Hopkins and Holben 2018 [35]	USA, OH, rural Appalachia (Athens)	Cross-sectional study	*Characteristics* *n* = 50 Ethnicity: 81.6% white Female: 67.4% Education: 46.9% college educated *Inclusion criteria* CG plot in Athens	To examine relationships among food security, produce intake and behaviors, health and social capital among community gardeners	All community gardeners (*n* = 120) in Athens invited		No intervention Individuals with CG plots	Survey distributed via email (response rate = 42%) Descriptive stats reported, no adjustment	Health and PA questions	100% ‘good’ to ‘excellent’ health at end of gardening season 66% do more PA due to CG No association of food security with PA
Litt et al. 2015 [38]	USA, CO, Denver	Cross-sectional survey	*n* = 469 *Characteristics* Age (mean ± SD): 46.1 ± 15.9y Female: 67.4% Education: 57.4% college educated 59.3% identified as gardeners *n* = 92 neighborhoods 49.6% residents college educated 25% residents minority 40.8% lived in area for ≥ 5 yrs *Inclusion criteria* English or Spanish speaking ≥ 18 yrs	To examine the direct and indirect pathways by which gardening influences self-rated health	Area-based sample of general population, *n* = 1154 randomly drawn from 40 block groups 13 gardens identified; List-based census of community gardeners *n* = 300		No intervention Individuals participating in CGs compared with non-gardeners	Surveys interviewer administered Path analysis controlling for age, education, years in neighborhood, % college education in neighborhood, observed incivilities	Self-rated health	Gardening did not predict self-rated health (β = 0.04, ns) Collective efficacy predicted higher self-rated health (β = 0.14, *p* < 0.05) Gardening impacted self-rated health indirectly, through social involvement, aesthetics and collective efficacy
Machida 2019 [39]	Japan	Cross-sectional survey	Web-based survey limited to age 60–69 y, professional farmers excluded (1) Community gardeners *n* = 129 Male n(%): 87(67%) Age (y): 64.1 ± 2.6 (2) Home gardeners (HG) *n* = 371 Male n(%):280(76% Age (y): 63.9 ± 2.7 (3) Non-gardeners *n* = 500 Male n(%): 327 (65%) Age (y): 63.3 ± 2.5	To study the relationship between community or home gardening and health status or a healthy lifestyle	The survey was conducted by a marketing company with 4.2 million people registered across all 47 prefectures in Japan		No INT	Odds Ratios adjusted for sex, age, family structure and employment status (not described)	BMI, exercise (> 30 min/d, at least 2 d/wk for over a year) and physically active (> 1 h/day), sitting time (categorized as < 3 h, 3–6 h and ≥ 6 h); walking speed faster than same generation and gender (yes/no) Sleep (enough + moderate versus not enough + shortage)	BMI (ref 20–24.9) (1) CG: Underweight (< 20): 0.97 (0.65, 1.46) Overweight or obese (≥ 25): 1.10 (0.78, 1.55) (2) HG: Underweight (< 20): 0.83 (0.46, 1.48) Overweight or obese (≥ 25): 0.69 (0.40, 1.19) Exercise (1) CG: 1.57 (1.19, 2.07) (2) HG: 1.79 (1.20, 2.67) PA (1) CG: 1.94 (1.45, 2.59) (2) HG: 2.32 (1.50, 3.59) Sitting time (ref ≥ 6 h/d) (1) CG 3–6 h/d: 1.59 (1.14, 2.22) < 3 h/d: 1.80 (1.21, 2.69) (2) HG: 3–6 h/d: 1.47 (0.91, 2.39) < 3 h/d: 1.74 (0.99, 3.05 Walking speed (faster than same generation and gender) (1) CG: 1.22 (0.92, 1.63) (2) HG: 1.48 (0.96, 2.26) Sleep (1) CG:0.99 (0.67, 1.46) (2) HG: 1.11 (0.63, 1.96)
Mangadu et al. 2017 [40]	USA, NM, US-Mexico border areas	Cross-sectional study	Two CGs accessible by public. (CG1, CG2) CG1 (*n* = 16) CG2 (*n* = 9) *Characteristics* % Male NR Age NR CG@ is a local government project comprising a neighborhood CG and a garden on a juvenile probation campus. Where possible, data from the probation campus are not extracted	To identify the best practices in implementing and increasing the potential or sustainability of community gardens	NR		NR	Descriptive statistics only. Not adjusted for anything	PA (1-item), ‘Do you think you are more physically active	Are you more physically active as a result of being engaged in CGs: CG1: Yes, *n* = NR (75%) CG2: yes, *n* = NR (100%)
Soga et al. 2017 [50]	Japan, Tokyo, Nerima district in central Tokyo	Cross-sectional survey	Gardeners (*n* = 165) vs non-gardeners (*n* = 167) *Characteristics* Gardeners: Male: 68.1% Age (mean ± SD): 62 ± 17y Non-gardeners: Male: 42% Age (mean ± SD): 61 ± 16y	To quantify effects of allotment gardening on physical, psychological and social health	Gardeners located by face-to-face recruitment at allotment gardens (90% response rate) Non-gardeners recruited via a letter sent to 1000 Nerima households (20% response rate)		NR	Adjusted for sex, age, household income, employment, smoking, drinking, vegetable intake and physical activity (days per week of > 30 min/day of moderate activity)	BMI (self-reported height, weight), Physical activity (days per week)	Compared with non-gardeners: Gardeners mean BMI (± SE) was 0.56 ± 0.39 higher Days of physical activity did not differ between gardeners (3.9 ± 2.3) and non-gardeners (3.9 ± 3.3)
Tharrey et al. 2020 [46]	France, Montpellier	Longitudinal cohort study Data collected at baseline and 1 year later	*Characteristics* (1) Community gardeners (*n* = 66) Male n(%): 16(24.2) Age (y): 44.0 ± 14.0 (2) Non-gardeners (*n* = 66) Male n(%): 16(24.2) Age (y): 44.9 ± 13.7 *Inclusion criteria* Starting gardening in a community garden; residents of Montpelier; ability to read French	To assess the impact or urban community garden participation the adoption of sustainable lifestyles	Gardeners recruited when new to the gardening community Non-gardeners recruited via volunteers for a population-based survey on food supply behaviors		Community gardens plots used collectively or individually	Analyzed with mixed-effects models with group by time interaction Adjustments for education, BMI, meals consumed outside the home, social desirability where appropriate	PA energy expenditure (PAEE), time spend inactive (< 1.5 METs) and moderate-to-vigorous activity (> 3 METs) using accelerometry worn for 9 consecutive days BMI from self-reported height and weight	PAEE at 1 year (mean ± SD) (1) 40.3 ± 12.3 (2) 39.9 ± 13.5 Inactivity at 1 year (h/day; mean ± SD) (1) 9.9 ± 1.5 (2) 9.8 ± 1.4 Moderate-to-vigorous activity at 1 year (h/d; mean ± SD) (1) 1.6 ± 0.7 (2) 1.7 ± 0.8 BMI at 1 year (mean ± SD) (1) 22.8 ± 3.1 (2) 23.9 ± 4.1
van den Berg et al. 2010 [51]	The Netherlands, “large cities”	Cross-sectional survey	Gardeners (*n* = 121) from 12 allotment gardens Non-gardener (*n* = 63) *Characteristics* Gardeners: Male: 53% Age (mean ± SD): 62 ± 12 y Non-gardeners: Male: 41% Age (mean ± SD): 56 ± 14 y	To directly compare the health, wellbeing and physical activity of allotment gardeners to that of controls without an allotment garden	Gardeners sent invitations to their home addresses Non-gardeners were responders living next to the home address of allotment gardeners		Ranged from residential parks, day-recreational parks and food production parks	Adjusted for age, sex, education, income, access to a garden at home, physical activity in winter and stressful life events, and included an age by gardening interaction term. Results separated by age	Physical activity as days/ week engaging in at least half an hour of intensive activities Count of chronic illnesses (e.g. cardiovas-cular, musculo-skeletal conditions) Count of GP consultations in past 2 months	Physical activity (days per week in summer): *Mean* ± *SD (unadjusted)* < *62 yrs* Gardeners 5.6 ± 0.2 Non-gardeners 5.1 ± 0.2 ≥ *62 yrs* Gardeners 5.8 ± 0.2 Non-gardeners 5.0 ± 0.2 *mean*_*adjusted*_ ± *SE* Chronic illness < 62 yrs Gardeners 0.6 ± 0.1 Non-gardeners 0.5 ± 0.1 ≥ 62 yrs Gardeners 0.5 ± 0.1 Non-gardeners 0.8 ± 0.2 GP consultations < 62 yrs Gardeners 0.7 ± 0.2 Non-gardeners 0.9 ± 0.2 ≥ 62 yrs Gardeners 0.5 ± 0.1 Non-gardeners 1.1 ± 0.2
Weltin 2013 [52] and Weltin and Lavin 2012 [48]	USA, IA, Dubuque	Pre-/post-	Immigrants from the Marshall Is living in Dubuque Iowa, who attended a local clinic for patients with diabetes (*n* = 17). Follow up data on *n* = 13 (*n* = 5 Gardeners *n* = 8 non-gardeners) *Characteristics* Male: 53% Age 33-81y (mean 51y)	To monitor HbA1c levels in Marshallese population who participated in a CG	From clinic		Clinic staff and their families donated supplies and taught how to prepare soil, plant, weed and harvest produce at a local church garden. Unclear if the garden was freely available for all to use	Comparison of pre-gardeners and non-gardeners using independent t tests. No adjustments	BMI, blood pressure and HbA1c levels 6 months after the interventions	*All mean* ± *SD* BMI Gardeners 30.2 ± 3.1 kg/m^2^ Non-gardeners 34.1 ± 1.4 kg/m^2^ Blood pressure NR separately for gardeners vs Non-gardeners HbA1c Gardeners 8.2 ± 1.6 Non-gardeners 9.3 ± 1.5
Zick et al. 2013 [53]	USA, UT, Salt Lake City	Cross-sectional study of linked administrative data	*n* = 198 community gardeners	To examine the association of participation in community gardening with healthy body weight	Wasatch CGs (WCG, non profit organization); Utah Population Database (UPDB) WCG staff provided details of 423 adults who gardened in 1 of WCG’s CG plots for ≥ 1 year; and not growing produce for sale 375 data linkage to UPDB, linkage rate of 88.7%		INT: community gardeners vs. 3 CON groups: (1) unrelated individuals who lived in gardeners’ neighborhoods, (2) siblings of community gardeners, and (3) spouses of the community gardeners	Multivariable analyses, controlling for year of BMI, age, gender, education, race	Self-reported height and weight (BMI)	*All mean* ± *SD* BMI Women CG vs women neighbors CG 23.9 ± 5.3 Neighbors 25.5 ± 5.7 BMI Women CG vs women siblings BMI: 23.9 ± 5.2 Siblings: 25.2 ± 5.6 Women CG vs women spouses CG: 24.3 ± 5.0 Spouses: 26.6 ± 12.8 Men CG vs neighbors CG: 24.7 ± 4.3 Neighbors 27.2 ± 4.8 Men CG vs Men siblings CG: 25.10 ± 4.63 Siblings: 25.63 ± 4.63 Men CG vs Men spouses CG: 25.34 ± 3.07 Spouses: 27.89 ± 5.83

*Abbreviations*: *BMI* Body mass index, *CG* Community garden, *COM* Comparison group, *CON* Control group, *F&V* Fruit and vegetable, *INT* Intervention group, *NR* Not reported, *OR* Odds ratio, *PA* Physical activity, *RCT* Randomized controlled trial, *SD* Standard deviation, *SE* Standard error, *SEP* Socioeconomic position

Weight-related outcomes were reported most frequently (11/16 (69%)) and the findings were mixed. Gardening was sometimes associated with lower weight-related outcomes, for example, there was less overweight and obesity among families participating in weekly gardening sessions [31]. However, studies also reported no difference in BMI, for example, in a cross-sectional survey of allotment gardeners compared with other active groups such as home gardeners and walkers [49], and a survey comparing gardeners to other local residents [50]. With respect to the five studies (5/16 (31%)) reporting blood pressure outcomes, a quasi-experimental study involving a non-randomized intervention suggested the odds of hypertension were lower [29] for gardeners compared with residents at a nearby county with no community garden, or that there were small or no differences in blood pressure [32, 48]. For other health outcomes such as physical activity, lung function, sleep and HBA1_c_ there were too few studies to synthesize evidence or outcomes were measured inconsistently or were inadequately powered to detect changes.

### Psychosocial outcomes

The 16 studies that reported psychosocial outcomes are summarized in Table 4. Seven of these studies were from the United States (44%), with two studies from Japan and the United Kingdom, and one study each from France, the Netherlands, Portugal, Singapore and Switzerland. The study designs were either cross-sectional surveys (12/16 (75%)), RCTs (2/16 (13%)), pre-/post (1/16 (6%)) or longitudinal (1/16 (6%)). Studies included between 20 [25] to 469 [38] participants. Outcomes were diverse, with community gardening associated with improvements in happiness [54], social support, social cohesion [35], mental health [50] and quality of life [55], as well as reductions in perceived stress [49]. In contrast, there were no differences observed in perceived health [38], although some outcomes such as effects on depression were not reported [33].

**Table 4:** Studies addressing psychosocial outcomes included in this review

First author, year	Country, setting	Study design	Sample characteristics (inclusion criteria, number, age and sex)	Aims	Sampling methods	Intervention / Community garden program	Data collection Analysis (including adjustments)	Outcomes	Results
Brown et al. 2020 [25]	USA, Montana, Native American community	RCT (1) Group-based Community gardening program (2) control (no gardening)	Native Americans with prediabetes or diabetes *N* = 20 Age (y): 15/20 were 45–64 years, 5/25 25–44 years Male n(%): 4/20 (25%) CON *n* = 12 INT *n* = 8	Determine feasibility of a group gardening program and potential for collecting health outcomes	Convenience sample of participant expressing an interest in the gardening study at a diabetes clinic	Raised beds for gardening chosen for proximity to college and health centre. Plus 10 × 90-min structured sessions with hands-on gardening and food preparation activities Outcomes measured at 7 months after baseline	Outcomes were reported as medians and ranges. Change from baseline was compared between the groups using Wilcoxon rank sum tests. No adjustments Sample numbers reported for each outcome as there was missing information for some outcomes	Quality of life (QOL), CES Depression Scale Tension-anxiety, depression-dejection, anger-hostility, vigour-activity, fatigue-inertia, and confusion-bewilderment (from Profile of Mood States Inventory—POMS)	QOL – psychological INT (*n* = 7) 0 [-3.3, 2.0] CON (*n* = 11) 0.2 [-4.0, 4.5] P = 0.772 QOL – social INT (*n* = 7) 0 [0, 2.0] CON (*n* = 11) 0.2 [-6.7, 4.0] P = 0.430 QOL – environment INT (*n* = 7) -1.0 [-1.5, 0.5] CON (*n* = 11) 0 [-0.5, 4.0] P = 0.013 QOL – physical INT (*n* = 7) -0.6 [-1.7, 0.6] CON (*n* = 11) -0.6 [-5.1, 2.9] P = 0.707 POMS – total mood disturbance INT (*n* = 8) -2.0 [-16, 18] CON (*n* = 9) 9 [(-1.0, 30] P = 0.049 POMS – tension anxiety INT (*n* = 8) − 0.5 (− 4.0 to 6.0) CON (*n* = 10) 1.0 (− 2.0 to 12.) P = 0.062 POMS – depression-dejection INT (*n* = 8) − 0.5 (− 4.0 to 6.0) CON (*n* = 10) 3.5 (− 2.0 to 19) P = 0.105 POMS – anger-hostility INT (*n* = 8) 0 (− 8.0 to 10) CON (*n* = 9) 6.0 (− 6.0 to 20) P = 0.180 POMS – vigor-activity INT (*n* = 8) − 3.0 (− 13 to 13) CON (*n* = 9) 0 (− 7.0 to 7.0) P = 0.382 POMS – fatigue-inertia INT (*n* = 8) − 2.5 (− 16 to 7.0) CON (*n* = 10) 2.0 (− 10 to 21) P = 0.246 POMS – confusion-bewilderment INT (*n* = 8) 0 (− 4.0 to 6.0) CON (*n* = 9) 2.0 (0 to 17) P = 0.119
Gerber et al. 2017 [56]	USA	Cross-sectional survey	Bhutanese community leaders recruited participants & collected data Bhutanese refugees in the USA who self-select as community gardeners (*n* = 22) or non-gardeners (*n* = 28) *Characteristics* Female: 62% Age (mean ± SD): 45 ± 15 yrs	To explore differences in indicators of distress and social support among Bhutanese refugees that participate in community gardens compared with those who do not	Bhutanese community events, word-of-mouth	Waiting list for plots. Families typically garden on one or two plots	Descriptive statistics only. No comparisons, & not adjusted for anything	Symptoms of post-traumatic stress disease, anxiety & depression using the Refugee Health Screener (15-items, score > 12 refer to mental health service) Patient Health Questionnaire (15-items, 3-point scale; cut-points 5, 10 & 15 indicate low, medium and high somatic symptoms) Perceived social support (Medical Outcomes Study Social Support Survey; 19-item, 5-point scale)	On average, more Gardeners lived in a house, and had lower medical bills, compared with non-gardeners Refugee Health Screener referrals (mean ± SD) Gardeners: 11.6 ± 9.2 Non-gardeners: 11.0 ± 9.9 Comparisons “not statistically different” Scores > 5 on Patient Health Questionnaire Gardeners: 14/22 (64%) Non-gardeners: 13/28 (46%) Gardeners experienced more somatic symptoms Standardized effect size (*d* = 0.36 95% CI -0.21, 0.91) Social support (mean ± SD) Gardeners: 61.3 ± 13.2 Non-gardeners: 52.5 ± 12.1 Gardeners reported more social support Standardized effect size (*d* = 0.70 95% CI 0.12, 1.27)
Grier et al. 2015 [57]	USA; Dan River, Virginia	Pre-/post	*n* = 43 *Characteristics* Ethnicity: 97.7% African American Age (mean) 8.7y Male: 46.5% Weight status: 34.1% overweight 18.2% obese *Inclusion criteria* Age: 5–17 y Child AND parent reside in housing authority full-time	To report on feasibility (demand, acceptability, implementation and limited-effectiveness) of a CG and nutrition education program	Two public housing authority sites – active members of the Dan River Partnership for a Healthy Community Adult site leaders knew families and youth; distributed recruitment material	Junior Master Gardener curriculum with nutrition focused lessons (informed by SCT). Weekly gardening sessions or gardening + nutrition education with site leaders	Interviewer administered survey Repeated measures ANOVA (ITT and complete case; ITT presented); effect sizes calculated	Psychosocial factors related to F&V consumption (not actual consumption) and nutrition knowledge	Increased self-efficacy for asking for F&V (ES: 0.39; *p* = 0.013) No change in willingness to try F&V (ES = 0.10; *p* = 0.310), self-efficacy for eating F&V (ES = 0.21; *p* = 0.119) or nutrition knowledge (ES = 0.10; *p* = 0.583)
Hawkins et al. 2011 [49]	UK, Wales, Cardiff	Cross-sectional study (1) Indoor exercise group (2) Walkers (3) Allotment gardeners (4) Home gardeners	*Characteristics* (1) *n* = 23 Age (y): 72.9 ± 6.9 Male: 3 (13%) (2) *n* = 25 Age (y): 62.4 ± 6.8 Male: 8 (32%) (3) *n* = 25 Age (y): 65.7 ± 9.1 Male: 17 (68%) (4) *n* = 21 Age (y): 69.5 ± 7.7 Male: 2 (10%) *Inclusion criteria* ≥ 50 y attending various local activity groups	Measure health status and perceived stress of allotment gardeners compared to other activity groups (indoor exercisers, walkers, home gardeners)	Recruited via leaflets, posters and visits to groups from researcher Response rate 87.8%	Compared leisure activity groups to members of allotment gardening group No intervention	Perceived stress: Cohen & Williamson 1988 Health-related Quality of Life, (Mental health component) using the SF-36v2 Social provisions Stress level btw groups adjusted for area-level SEP	Perceived stress	Significantly lower perceived stress among allotment gardeners than other activity groups Perceived stress (mean ± SD) (1) 15.8 ± 6.1 (2) 13.6 ± 5.4 (3) 9.8 ± 5.8 (4) 12.0 ± 4.8 QOL Mental health (median, IQR) (1) 50 (46.6–54.4) (2) 56.1 (51.7–58.4) (3) 55.3 (50.1–58.6) (4) 55.8 (50.7–58.8)
Hartwig and Mason 2016 [33]	USA, MN, Twin Cities	Cross-sectional surveys	*n* = 97 *Characteristics* Female: 65% English: 18% good/fluent Age (mean): 39y (16–80 y) Ethnicity: 67% Karen (Burmese)	To evaluate church CGs serving refugee and immigrant populations, reporting primary health and social benefits	All gardeners at 8 gardens invited (Response rate = 44–45%) Gardens purposively sampled based on: - 2 yrs participation - # gardeners - primary language of gardeners	8 church gardens serving refugees and immigrants	Measured early and late season harvest (Jul-Sept) Descriptive stats used: change in mean/% early and late season No adjustments	Depression Gardening alone/with others (social interaction)	Change in depression risk not reported (12% reached cut-off for additional screening) Frequency of social interactions declined from early to late season harvest
Heilmayr and Friedman, 2020 [34]	USA, CA	RCT with 5 INT groups: (1) Community gardening (2) moderate indoor exercise (3) Exposure to nature (4) Social club (watching films) (5) Indoor container gardening	University students Baseline data reported in combination (not by group allocation) Age (y): 20.6 ± 3.3 Male: 31.2% (1) *n* = 21 (2) *n* = 21 (3) *n* = 23 (4) *n* = 22 (5) *n* = 23	To compare community gardening with four theoretically driven comparison groups to understand possible causal mechanisms around how community gardens have improved outcomes	Convenience sample recruited via flyers, emails and the Psychology Subject Pool	4 week INT; assigned an activity for 2–3 h/wk	Data were analyzed by ANOVA with pre-/post-test values to assess how groups changed from baseline and a group by time interaction	Emotional wellbeing (Comprised of: Perceived stress, Happiness, Self-efficacy, Positive and Negative Affect) Social relationships (Comprised of: Companionship, Social integration)	Emotional wellbeing (post-test only; mean ± SD) (1) 65.4 ± 14.8 (2) 66.6 ± 15.5 (3) 66.1 ± 13.3 (4) 63.6 ± 15.5 (5) 67.1 ± 14.4 Social relationships (post-test only; mean ± SD) (1) 62.3 ± 10.8 (2) 63.5 ± 11.9 (3) 59.3 ± 14.1 (4) 59.3 ± 16.6 (5) 60.7 ± 11.4
Hopkins and Holben 2018 [35]	USA, OH, rural Appalachia (Athens)	Cross-sectional study	-*n* = 50 Inclusion criteria: CG plot in Athens *Characteristics* Ethnicity: 81.6% white Female: 67.4% Education:46.9% college educated	To examine relationships among food security, produce intake and behaviors, health and social capital among community gardeners	All community gardeners (*n* = 120) in Athens	No INT Individuals with CG plots	Survey distributed via email (Response rate = 42%) Descriptive stats reported, no adjustment	Social capital (made new friends)	Social cohesion 74% have made new friends due to CG No association of food security with social capital
Koay et al. 2020 [58]	Singapore	Cross- sectional survey	(1) Community gardener *N* = 45 Male n (%): 25(56%) Age (mean ± SD): 60.2y (± 13.3) Ethnicity: 40 (89%) Chinese Education: 18(40%) Tertiary (2) Home gardener *N* = 38 Male n(%) 6(84%) Age (mean ± SD): 43.8 ± 13.0 Ethnicity: Chinese 35(92%) Education: Tertiary 33(87%) (3) Non-gardening control *N* = 28 Male n (%) 12(43%) Age (mean ± SD): 55.5 ± 11.6 Ethnicity: Chinese 23(82%) Education: Tertiary 13(46%)	Study relationship between community gardening and mental health benefits	Snowball recruitment from gardens and outdoor activity groups	Community in Bloom program of government supported	Multivariate ANCOVA with adjustment for age and connection to nature	Perceived stress scale (10-item, 5-point scale) Personal Wellbeing Index (7-item, 11 point scale) Brief resilience scale (6-item, 5-point scale)	Perceived stress scale (mean ± SD) (1) 11.4 ± 6.4 (2) 15.5 ± 6.1 (3) 7.0 ± 0.8 Personal wellbeing index (mean ± SD) (1) 8.2 ± 1.1 (2) 7.0 ± 1.2 (3) 7.0 ± 0.8 Brief resilience scale (mean ± SD) (1) 3.7 ± 0.7 (2) 3.5 ± 0.6 (3) 3.0 ± 0.8
Litt et al. 2015 [38]	USA; Denver, Colorado	Cross-sectional survey	*n* = 469 *Characteristics* Age (mean): 46.1y (± 15.9) Female: 67.4% Education: 57.4% college educated Identified as gardeners: 59.3% *Inclusion criteria* English or Spanish speaking, ≥ 18yrs	To examine the direct and indirect pathways by which gardening influence self-rated health	Area-based sample of general population *n* = 1154 randomly drawn from 40 block groups 13 gardens identified; List-based census of community gardeners *n* = 300	No intervention Individuals participating in CGs compared with non-gardeners	Surveys interviewer administered Path analysis controlling for age, education, years in neighborhood, % college education in neighborhood, observed incivilities	Social involvement Collective efficacy Neighborhood attachment	Path model results: Data fit model adequately, accounting for 22% variance in self-rated health and 4% in F&V intake Gardening predicted social involvement (β = 0.36; *p* < 0.001) Social involvement (β = 0.11, *p* < 0.05) and aesthetics (β = 0.46, *p* < 0.001) predicted Collective efficacy Collective efficacy predicted neighborhood attachment (β = 0.29, *p* < 0.001)
Machida 2019 [39]	Japan	Cross-sectional survey	Web-based survey limited to age 60–69 y, professional farmers excluded (1) Community gardeners *n* = 129 Male n (%): 87(67%) Age (y): 64.1 ± 2.6 (2) Home gardeners (HG) *n* = 371 Male n(%):280(76% Age (mean ± SD): 63.9y ± 2.7 (3) Non-gardeners *n* = 500 Male n(%): 327 (65%) Age (mean ± SD): 63.3y ± 2.5	To study the relationship between community or home gardening and health status or a healthy lifestyle	The survey was conducted by a marketing company with 4.2 million people registered across all 47 prefectures in Japan	NA	Odds Ratios adjusted for sex, age, family structure and employment status (not described)	Happiness (single item, 11-point scale) dichotomized to ≤ 6 vs ≥ 7 Psychological distress using 4 items of the K6 (4-point scale) dichotomized at ≤ 8 vs ≥ 9	Happiness (1) CG: 1.60 (1.18, 2.16) (2) HG: 0.89 (0.59, 1.34) Distress (1) CG: 0.85 (0.57, 1.27) (2) HG: 0.72 (0.38, 1.36)
Mourao et al. 2019 [54]	Portugal	Cross-sectional survey	Invitation from the Urban Allotment Garden office, sent to 30 gardeners per session. Six sessions performed, resulting in 65 validated responses Lived in urban areas of the council, 90.8% *Characteristics* Male: 56.9% Age: 26–45 yrs: 36.9% 46–65 yrs: 47.7% > 65 yrs: 15.4%	To evaluate the happiness and well-being of the Portugal population, based on the urban organic allotment gardens	Self-administered questionnaires	Permanent resident, garden a family plot	Pearson correlation. No adjustment	Personal wellbeing scale Subjective happiness scale	Gardening frequency: Once a week: 10.5% Few days a week: 47.7% Daily: 41.5% Degree of life satisfaction (personal well-being index): Mean 74.5% (0–100%) Greater frequency to gardens was associated with higher perspective of subjective happiness and compared to their peers
Soga et al. 2017 [50]	Japan, Tokyo, Nerima district in central Tokyo	Cross-sectional survey	Gardeners (*n* = 165) vs non-gardeners (*n* = 167) *Characteristics* Gardeners: Male: 68.1% Age (mean ± SD): 62 ± 17y Non-gardeners: Male: 42% Age (mean ± SD): 61 ± 16y	To quantify effects of allotment gardening on physical, psychological and social health	Gardeners located by face-to-face recruitment at allotment gardens (90% response rate). Non-gardeners recruited via a letter sent to 1000 Nerima households (20% response rate)	No INT	Adjusted for sex, age, household income, employment, smoking, drinking, vegetable intake and PA (days per week of > 30 min/day of moderate activity)	Mental health using 12-item General Health questionnaire (scores 0–12)	Mental health Compared with non-gardeners, mean mental health scores for gardeners (± SE) was -0.91 (0.42) higher (*P* < 0.05), indicating improvements in mental health
Swami 2020 [59]	UK, London	Cross-sectional survey	English-speaking adults. One participant per allotment (1) allotment gardeners (*n* = 84) (2) non-gardeners (*n* = 81) Full sample Male (%): 40% Age (mean ± SD): 44.7y ± 18.2	To examine the effect of allotment gardening on state body image	Gardeners recruited from 12 allotment sites via direct approach. Non-gardeners recruited from supermarkets closest to the allotment sites. They could no “do anything in the garden”	Not described	Non-gardeners ‘matched’ to gardeners but no description of matching process or characteristic. Analysis by unpaired t-test with no adjustments (including no adjustment of matching criteria) Bonferroni correction of p values	State body image using a 10 cm visual analogue scale Body Appreciation Scale-2 (10-items, 5-point scale) Functionality Appreciation Scale (7-item, 5-point scale) Authentic Pride subscale of the Body and Appearance Self-Conscious Emotions Scale (6-items, 5-point scale)	Body appreciation (mean ± SD) (1) 3.5 ± 0.8 (2) 3.1 ± 0.8 Functionality appreciation (mean ± SD) (1) 3.5 ± 0.7 (2)3.2 ± 0.8 Body pride (mean ± SD) (1) 3.1 ± 0.9 (2) 2.6 ± 0.9
Tharrey et al. 2020 [46]	France, Montpellier	Longitudinal cohort study Data collected at baseline and 1 year later	*Characteristics* (1) Community gardeners (*n* = 66) Male n(%): 16(24.2) Age (y): 44.0 ± 14.0 (2) Non-gardeners (*n* = 66) Male n (%): 16(24.2) Age (y): 44.9 ± 13.7 *Inclusion criteria* Starting gardening in a CG; residents of Montpelier; ability to read French	To assess the impact or urban community garden participation the adoption of sustainable lifestyles	Gardeners recruited when new to the gardening community Non-gardeners recruited via volunteers for a population-based survey on food supply behaviors	Community gardens plots used collectively or individually	Analyzed with mixed-effects models with group by time interaction Adjustments for education, BMI, meals consumed outside the home, social desirability where appropriate	Warwick-Edinburgh Mental Wellbeing Scale (WEMWBS; 14-item, 5-point scale) Loneliness scale v3 (20-items 4-point scale)	Wellbeing at 1 year (mean ± SD) (1) 51.5 ± 6.9 (2) 51.5 ± 5.7 Loneliness at 1 year (mean ± SD) (1) 40.1 ± 10.9 (2) 40.5 ± 9.5
van den Berg et al. 2010 [51]	The Netherlands, “large cities”	Cross-sectional survey	Gardeners (*n* = 121) from 12 allotment gardens Non-gardener (*n* = 63) *Characteristics* Gardeners: Male: 53% Age (mean ± SD): 62 ± 12 y Non-gardeners: Male: 41% Age (mean ± SD): 56 ± 14 y	To directly compare the health, wellbeing and physical activity of allotment gardeners to that of controls without an allotment garden	Gardeners sent invitations to their home addresses Non-gardeners were responders living next to the home address of allotment gardeners	Ranged from residential parks, day-recreational parks and food production parks	Adjusted for age, sex, education, income, access to a garden at home, PA in winter and stressful life events, and included an age by gardening interaction term. Results separated by age. For all outcomes	Stress in past month (2-items, 6-point scale), Life Satisfaction Index (8-item, 3-point response) Loneliness (2-items, 0–1 responses) Social contacts (2-items, scores range 1–12)	*All mean*_*adjusted*_ ± *SE* Stress < 62 yrs Gardeners 3.2 ± 0.1 Non-gardeners 2.9 ± 0.2 ≥ 62 yrs Gardeners 2.1 ± 0.1 Non-gardeners 2.5 ± 0.2 Life satisfaction < 62 yrs Gardeners 2.2 ± 0.1 Non-gardeners 2.2 ± 0.1 ≥ 62 yrs Gardeners 2.3 ± 0.1 Non-gardeners 2.0 ± 0.1 Loneliness < 62 yrs Gardeners 0.7 ± 0.1 Non-gardeners 0.6 ± 0.1 ≥ 62 yrs Gardeners 0.3 ± 0.1 Non-gardeners 0.8 ± 0.2 Social contacts < 62 yrs Gardeners 6.1 ± 0.4 Non-gardeners 7.0 ± 0.5 ≥ 62 yrs Gardeners 8.1 ± 0.4 Non-gardeners 6.2 ± 0.7
Young et al. 2020 [60]	Switzerland, Zurich	Cross-sectional survey	Materials provided in 4 languages used locally. Limited to one person per allotment (1) Allotment gardeners (*n* = 108) Male (%): 52% Age (y): 59 (SD NR) (2) Domestic gardeners (*n* = 193) Male (%): 33% Age (y): 54 (SD NR)	To identify whether gardening is a source of stress (i.e. stress as a result of the garden)	Allotment gardeners drawn in a two-stage probabilistic sampling strategy (response rate 48%.) Domestic gardeners drawn from a random sample of individuals living in Zurich (response rate 27%)	Allotments typically 100–200 m2, with rules to prohibit invasive species and construction on site. Domestic gardens are available to householders who can afford to buy/rent a residence with a garden (~ 10% of population)	Independent t-test Structural equation model (SEM) with robust standard errors, full information maximum-likelihood for missing data and adjustment for age, gender, employment, job type and biodiversity preference	Single question “I often feel under pressure when I think of the tasks that need doing in my garden” (5-point response)	Garden-related stress (mean ± SD) (1) 2.2 ± 1.2 (2) 2.5 ± 1.1 Allotment gardeners reported lower stress than domestic gardeners (β = -0.167, *p* = 0.013) when controlling for socioeconomic variables in SEM

*Abbreviations*: *CG* Community garden, *CI* Confidence interval, *COM* Comparison group, *CON* Control group, *ES* Effect size, *F&V* Fruit and vegetable, *INT* Intervention group, *ITT* Intention-to-treat, *NR* Not reported, *OR* Odds ratio, *PA* Physical activity, *RCT* Randomized controlled trial, *SD* Standard deviation, *SE* Standard error, *SEP* Socioeconomic position

### Community outcomes

Table 5 summarizes the seven studies that reported community outcomes. Three of these studies were conducted in the United States, with one each from Canada, Japan, Portugal and the Netherlands. All were cross-sectional in design and sample sizes ranged from 25 [40] to 500 [39]. Findings were generally positive for gardening and community-related outcomes. For example, gardeners had higher neighborhood attachment [61], perceptions of neighborhood aesthetics [38], measures of social cohesion [50] and civic participation [42], compared with non-gardeners, although Machida et al. did not report greater connection among neighbors among community gardeners compared with non-gardeners [39].

**Table 5:** Studies addressing community outcomes included in this review

First author, year	Country, setting	Study design	Sample characteristics (inclusion criteria, number, age and sex)	Aims	Sampling methods	Intervention / Community garden program	Data collection Analysis (including adjustments)	Outcomes	Results
Comstock et al. 2010 [61]	USA, CO, Denver	Cross-sectional survey of local neighborhood	*N* = 410 *Inclusion criteria* Living in area identified for sampling and ≥ 18 y	To compare people who participate in community and home gardening activities with people who do not garden	Area(block)-based probability sampling of general population (*n* = 1154), & list-based census of community gardeners (*n* = 300) 473 household respondents but 410 in analysis	No INT	Hierarchical linear models adjustment for: years living in neighborhood, own home, ethnicity, education, incivilities, safety, efficacy, gardener or not, local block characteristics (college degree, crime, collective efficacy, incivilities)	Neighborhood attachment 6 questions, 4-point Likert scale ranging from 1 strongly disagree to 4 strongly agree	59% response rate (473 respondents/1454 households attempted to contact) 8% community gardeners (31/410 respondents) Neighborhood attachment (Standardised beta, no SD or CI reported) Community gardener compared with non-gardener β = 0.23, *p* < 0.05
Litt et al. 2015 [38]	USA, CO, Denver	Cross-sectional survey	= 469 *Characteristics* Age (mean): 46.1y (± 15.9) Female: 67.4% Education: 57.4% college educated Identified as gardeners: 59.3% *Inclusion criteria* English or Spanish speaking, ≥ 18yrs	To examine the direct and indirect pathways by which garden influence self-rated health	Area-based sample of general population *n* = 1154 randomly drawn from 40 block groups 13 gardens identified; List-based census of community gardeners *n* = 300	No intervention Individuals participating in community gardens compared with non-gardeners	Surveys interviewer administered Path analysis controlling for age, education, yrs in neighborhood, % college education in neighborhood, observed incivilities	Neighborhood aesthetics	Gardening predicted neighborhood aesthetics (β = 0.35, *p* < 0.001)
Machida 2019 [39]	Japan	Cross-sectional survey	Web-based survey limited to age 60–69 y, professional farmers excluded (1) Community gardeners *n* = 129 Male n (%): 87(67%) Age (mean ± SD): 64.1y ± 2.6 (2) Home gardeners *n* = 371 Male n (%):280 (76% Age (mean ± SD): 63.9y ± 2.7 (3) Non-gardeners *n* = 500 Male n (%): 327 (65%) Age (mean ± SD): 63.3y ± 2.5	To study the relationship between community or home gardening and health status or a healthy lifestyle	The survey was conducted by a marketing company with 4.2 million people registered across all 47 prefectures in Japan	NA	Odds Ratios adjusted for sex, age, family structure and employment status (not described)	Connection with neighbors (≥ moderate vs ≤ little)	Connection with neighbors (1) CG: 2.08 (1.53, 2.82) (2) Home gardeners: 2.03 (1.33, 3.09)
Mangadu et al. 2017 [40]	USA, NM, US-Mexico border areas	Cross-sectional study	Two CGs accessible by the public. (CG1, CG2) CG1 (*n* = 16) CG2 (*n* = 9) *Characteristics* % Male NR Age NR CG@ is a local government project comprising a neighborhood CG and a garden on a juvenile probation campus. Where possible, data from the probation campus are not extracted	To identify the best practices in implementing and increasing the potential or sustainability of community gardens	NR	NR	Descriptive statistics only. Not adjusted for anything	Single question: I am more involved in this neighborhood?	I am more involved in this neighborhood CG1: Yes, *n* = 16 (100%) CG2: yes, *n* = 4 (44%)
Roncarolo et al. 2015 [42]	Canada, Montreal	Cross-sectional study	Participants sampled from 16 traditional (e.g. food banks, *n* = 711) or 6 alternative (e.g. community gardens) venues (*n* = 113) *Characteristics* Female: 55% Age: 52% aged 30-49y	To compare outcomes between users of traditional versus alternative organizations	Sampled from food security organizations with ≥ 50 new members (traditional) or ≥ 30 new members (alternative)	Not precisely described but indicated as being organizations (gardens) that nurture solidarity, and have goals of reducing social inequalities	Multilevel logistic regression to account for clustering by study site. Adjusted for sex, country of birth, marital status, employment, education, income and number of people in the household	Civic participation (user / volunteer/ none)	Civic participation None = reference User OR_adjusted_ = 1.17 (0.60, 2.25) Member OR_adjusted_ = 2.21 (1.10, 4.45)
Soga et al. 2017 [50]	Japan, Tokyo, Nerima district in central Tokyo	Cross-sectional survey	Gardeners (*n* = 165) vs non-gardeners (*n* = 167) *Characteristics* Gardeners: Male: 68.1% Age (mean ± SD): 62 ± 17y Non-gardeners: Male: 42% Age (mean ± SD): 61 ± 16y	To quantify effects of allotment gardening on physical, psychological and social health	Gardeners located by face-to-face recruitment at allotment gardens (90% response rate). Non-gardeners recruited via a letter sent to 1000 Nerima households (20% response rate)	NR	Adjusted for sex, age, household income, employment, smoking, drinking, vegetable intake and PA (days per week of > 30 min/day of moderate activity)	Social cohesion using the Social Cohesion and Trust Scale (X items, 5-point scale)	Compared with non-gardeners, gardeners mean social cohesion scores (± SE) were 1.57 (0.57) higher (*P* < 0.001)
Veen et al. 2016 [47]	The Netherlands	Cross-sectional	7 gardens (6 completed questionnaire) *N* = 237 respondents *Inclusion criteria* NR	To investigate the extent to which community gardens influence the enhancement of social cohesion	Gardens selected to ensure homo- and heterogeneity in neighborhood, plot type and harvest consumption type Recruitment via newsletter and letter to CGs	No INT Membership at one of selected community gardens	F-statistic, generalized linear models, chi-square No adjustments	Social cohesion (importance of garden socially)	Individual gardeners vs communal gardeners at CGs; NS for social cohesion

*Abbreviations*: *CG* Community garden, *CI* Confidence interval, *NR* Not reported, *OR* Odds ratio, *SD* Standard deviation, *SE* Standard error

### Effects of community gardens according to location or SEP

No studies were identified that directly compared effects in different locations or by socioeconomic position (SEP).

### Characteristics of community gardeners

We located 24 studies that described users of community gardens (Table 6). Studies were from cross-sectional surveys (23/24 (96%)) except for one longitudinal study. Sample sizes ranging from 37 [62] to 1916 [63]. Seven studies were from the United States (8/24 (33%)), with two studies each from Canada and the Netherlands, and one each from Australia, Czechia, Denmark, Germany, Israel, Italy, Japan, Nigeria, Portugal, Spain, South Africa and Zimbabwe. Of these studies, 16 (67%) made no comparisons to non-gardeners and therefore little inference can be made from these studies but they have been tabulated for completeness. Of the eight studies (33%) that compared community gardeners against some other community group (such as non-gardeners or home gardeners), some reported that gardeners had higher educational attainment and income [42, 64] although this was not consistent as other studies reported no differences [50, 51]. Gardeners also tended to be older or were retirees [50, 51, 65].

**Table 6:** Characteristics of individuals or households who use community gardens

First author, year	Country, setting	Study design	Sample characteristics (inclusion criteria, number, age and sex)	Study aims	Sampling methods	Intervention / Community garden program	Data collection, analysis (including adjustments)	Results
**Studies describing characteristics of gardeners (no comparison against other groups)**								
Algert et al. 2016 [28]	USA, Calif., San Jose	Cross-sectional survey	Two groups: *Characteristics* Community gardeners: *n* = 85 Female: 84% Age (mean ± SD): 49y (± 13) Home gardeners *n* = 50 Female: 50% Age (mean ± SD): 58 (± 12) y	To compare whether the two groups of gardeners (community and home) increased their vegetable intake while gardening	1) CG: Face-to-face recruitment at 4 separate allotments 2) La Mesa Verde (LMV): Recruited through existing home gardening project for low-income families Response rate not reported	Participants in 1) San Jose’s Community Garden program which provides space to grow food, socialize and learn about gardening) 2) Local govt. funded (LMV; home gardening project) which provides raised beds, soil, seeds and plants; instruction on organic gardening workshops	Demographic characteristics of community gardeners No adjustment	Community gardeners only - Low income than median income in county - 56% had college-level education - 53% white race - 66% lived in a house (not apartment) - mean BMI 26.3 (± 5.3)
Bussell et al. 2017 [66]	USA, San Diego	Cross-sectional survey	120 community gardeners at 8 rural and urban sites *Characteristics* Age: 76.6% aged 30–79 yrs	To determine the reasons why people pursue community gardening and to discern whether low-income community gardeners are motivated by perceived or actual economic benefits	88 CGs located throughout the region but primarily in urban areas, with significant number located in low-income communities	Larger, more mature CGs as well as younger and smaller gardens	Reasons why people use CG, including social, well-being and economic reasons; questions about types and volume of produce commonly grown; adequacy of the CGs in meeting needs of gardeners	Motivations for CG: - 84% to grow food - 60% to improve health - 39% to make new friends - 50% community connections are benefit of belonging to a CG - 61% made new friendships - 65% relaxing - 79% spending time outdoors - 90% improve diet - 90% confirmed that their household had eaten more fresh F&V since started growing own produce Ethnicity: 40% Caucasian 23.3% Hispanic or Latino 6.7% African-American 7.5% Asian 6.7% African 5% Middle Eastern 5% other ethnicities 51% with ≥ 3 people in household 36.7%, retired 16.6% bachelor or postgraduate degree 45% high school degree but no further education
Edeoghon and Okoedo-Okojie 2015 [67]	Nigeria, Lagos State	Cross-sectional survey	Youths involved in urban agriculture *N* = 140 Male: 51% Age: < 20 yrs: 17% 21–30 yrs: 39% 31–40 yrs: 33% 41–50 yrs: 11%	To examine socio-economic characteristic of study respondents	Chose 3/5 wards where intensive urban agriculture is practices. Selected farmers attending those settings	NR	Sociodemographic characteristic of people who use CGs No comparisons, & not adjusted for anything	Marital status Single: 35% Married: 57% Divorced: 9% Education No formal: 1% Primary: 7% Junior secondary: 6% Snr secondary: 44% Other: 29% Degree: 12% Employment Yes: 29% No: 71% Household size < 3 people: 32% 3–6 people: 51% > 6 people: 17%
Dubova and Machac 2019 [62]	Czechia, Kuchyňka and Vidimova	Cross-sectional survey	Inclusion criteria not reported (1) Kuchyňka *n* = 13 respondents / 23 users (2) Vidimova *n* = 24/45 members	To understand garden users perceptions of benefits and social benefits	Convenience sample of garden users	Kuchyňka garden is terraced vegetable beds where goal is vegetable independence. Vidimova garden has mobile garden beds and hosts cultural activities	NR	Data reported as text Respondents were more likely to be female, aged 31–40 years, 1 child, university degree (numbers not reported)
Egerer et al. 2019 [68]	Australia, Melbourne	Cross- sectional survey	Adult users of urban community gardens (11 gardens) *n* = 189 Male n (%):82 (43%)	To understand the importance of community gardens to users	Recruited via “intercept sampling”, a method of sample of garden users (convenience sampling)	Not-for-profit local spaces to grow fresh food, practice sustainability, build food literacy and skills, build community connection	Descriptive analysis No adjustments. No comparison group	Speaks English n(%) 146 (77%%) English as second language n (%): 36 (19%) Not born in Australia *n* = 62 (33%)
Filkobski et al. 2016 [69]	Israel	Cross-sectional survey	Participants in CGs located in all the big cities of Israel, medium size towns and rural settlements as well as different types of programs that exist in urban community gardening *N* = 44 Age: 11–20 yrs: 20.9% 71–90 yrs: 11.6%	To explore the extent and characteristics of CGs in Israel and the local public’s involvement in these projects	136 CG coordinators via email, or at conference and training for CGs Response rate, 32%	Fenced and non-fenced CGs	Questionnaire sent to CG coordinators across the country to explore general characteristics of Israeli community gardens Survey questionnaire on garden location, previous site conditions physical features, profile of participants, sources of support and funding, objectives and activities	Users of the gardens Families with young children 58.5% Religion Jewish: 91% Muslim: 9% Geographic origins Born in Israel: 50.8% Immigrants from Ethiopia: 20.6% Former USSR (14.3%): USA: 7.9% Income level Average: 36.2% Below average: 34.5%
Gauder et al. 2019 [70]	Germany, multiple regions (66 cities and 9 states)	Cross-sectional survey	Details NR *n* = 173 Male (%): 25% Age (y) n (%) 20–29: 24% 30–39: 29% 40–49: 16% 50–59: 17% ≥ 60 y: 13%	To characterize participants of self-harvest gardens	Recruited online. Providers of self-harvest gardens (*n* = 95) were contacted and asked to forward survey to their participants	Self-harvest gardens where providers chose and plant the vegetable crops, provides advice, water and tools. Gardeners carry out watering, weeding and harvesting for personal use	Descriptive analysis No adjustments. No comparison group	Schooling (*n* = 173) Secondary n(%): 2% Professional: 14% Qualified for University: 62% Degree: 18% Promotion/habilitation: 1% Occupation (*n* = 173) Employed: 66% Student: 16% Retired: 8% Self-employed: 8% Homemaker: 2% Job training: 1% Relationship (*n* = 173) Married: 45% In a relationship: 38% Single: 18% Parents: 53% Lived in area > 5 years: 75%
Grebitus et al. 2017 [71]	USA, AZ, Arizona State University (class not named)	Cross sectional survey	Undergraduate university students (*n* = 325) who were given 1% credit for completing survey *Characteristics* Female: 38% Age (mean ± SD): 23 ± 4 y	To investigate the impact of consumer perception, knowledge and attitudes towards the likelihood to grow own produce at urban farms	Online survey available to students taking a course at Arizona State University	No program. Study is about the *likelihood* of growing food on urban farms	Descriptive statistics extracted. Likelihood to grow produce at an urban farm (1-item, 7-point scale) Analysis not adjusted for other variables	44% likely to grow their own produce at urban farms Participants that were likely to grow their own produce were more likely to be female, older, more educated, purchase foods locally and have knowledge about urban agriculture
Grubb and Vogel 2019 [72]	USA, Minnesota, Minneapolis and St Paul	Cross-sectional survey	Urban farms, youth gardens, ornamental gardens and those outside the area were excluded (101 gardens included) *Characteristics* *N* = 181 Male n(%): 45 (25%) Age (y): mean 48.4; median [IQR] 48 [34, 62]	To understand relationships between urban gardening and food literacy among adults	Snowball sampling by emailing community garden coordinator to pass on online survey	CGs defined as people who garden collectively on a plot and live in an urban area	Descriptive analysis No adjustments. No comparison group	Education n (%) High school/GED: 15(8%) College: 21(12%) Degree: 82(45%) Masters or higher: 63(35%) Rural upbringing 57(32%) Gardener type Food: 173(96%) Ornamental: 8(4%)
Langemeyer et al. 2018 [73]	Spain, Barcelona 27 urban gardens	Cross-sectional survey	Home or school gardens excluded *N* = 201 About three quarters of urban gardeners in Barcelona were male, 80% were aged > 50 y	To uncover key enabling factors for ecosystem services	NR	NR	Descriptive statistics extracted	70% retired 40% had education beyond secondary school (compared with 20% for all of Catalonia). 39% were Catalonian, 54% Andalucía and 6% from other European or non-European countries
Migliore et al. 2019 [74]	Palermo, Sicily, Italy	Cross-sectional survey	*Characteristics* Gardeners (*n* = 176) Male (%): 74(42%) Age n (%): 21–34 y: 18 (10%) 35–45 y: 31 (18%) 46–55 y: 44 (32%) 56–65 y: 56 (32%) 66–76 y: 27 (58%) *Inclusion criteria* NR	To understand citizens motivations for participating in Cgs	Convenience sample from 6 of the 7 gardens in the city, comprising 75% of the gardeners at those sites	NR	Face-to-face survey	Education n(%) Primary 8 (5%) Lower secondary 29 (17%) Upper secondary 68 (39%) University degree or higher 71 (40%) Income (Euros) < 1,500: 18 (10%) ~ 2000: 43 (24%) ~ 2,500: 55 (31%) > 3,000: 27 (15%) No answer: 33 (19%) Household members n (%) 1: 23 (13%) 2: 39(22%) 3: 46 (26%) 4: 52 (30%) 5: 14 (8%) > 5: 2 (1%)
Mourao et al. 2019 [54]	Portugal	Cross-sectional survey	Invitation from the Urban Allotment Garden office, sent to 30 gardeners per session. Six sessions performed, resulting in 65 validated responses *Characteristics* Male, 56.9% Age group (y) 26–45: 36.9% 46–65: 47.7% > 65: 15.4%	To evaluate the happiness and well-being of the Portugal population, based on the urban organic allotment gardens	Self-administered questionnaires	Permanent resident, garden a family plot	Personal wellbeing scale Subjective happiness scale Pearson correlation. Analysis not adjusted for other variables	Demographics Married: 72% Higher than year 12: 56.9% Working: 46.2% Unemployed: 21.5% Retired: 32.3% Monthly income < €500: 16.9% €500–1250: 47.8% > €1250: 35.3% Housing Independently housed: 26% Apartments: 56% Lived in urban council area 90.8% Gardening frequency Once a week: 10.5% Few days a week: 47.7% Daily: 41.5%
Roberts and Shackleton 2018 [75]	South Africa, Eastern Cape	Cross-sectional survey	*N* = 69 gardeners *Characteristics* Male: 51% male Age (mean ± SD): 56y ± 18	To understand the nature of community gardening in poor provinces	Gardeners on site at 4 randomly selected gardens per town	Spaces for food production	Descriptive statistics only. Not adjusted for anything	*All mean* ± *SD* Years of education 7.7 ± 3.8 Household size 6.1 ± 2.6 Number of social grants household receives 1.4 ± 1.3
Spliethoff et al. 2016 [45]	New York City (NYC), USA	Written survey Cross-sectional	NYC community gardeners *Characteristics* *n* = 46 (information on a total of 93 adults and 13 children in their households) Age: NR *Inclusion criteria* NR	To assess vegetable consumption rates and time spent in the garden in NYC community gardeners	Mailing to contact gardeners at 76 NYC community gardens from which soil had been sampled (separate aim) and to volunteers at NYC gardening workshops	CG vs nationally representative non-gardeners	Median and 95th percentile consumption rates for crops (fruiting, leafy, root, and herb) for gardeners (*n* = 46), compared with other household members (18 + y; *n* = 47) Mann–Whitney U test for comparing total vegetable intake in mg/kg body weight/day	Description of crop grown in past 12 months and estimate crop harvested during that time; estimate fractions of harvest consumed/not consumed by themselves plus by household; age, body weight; servings of F&V
Veen and Eiter 2018 [76]	Netherlands	Cross-sectional survey	Almere, Netherlands Found by volunteering to write gardener “portraits” for the allotment magazine; the editor of the magazine recruited the interviewees *N* = 81 Age group (y) 25–34: 1% 35–44: 12% 45–54: 19% 55–64: 38% ≥ 65: 30%	To explore differences in motivation for and actual use of allotment gardens	Received the questionnaire on paper, by general mail, including a stamped return envelope	Waiting list for plots. Gardeners can cultivate more than one plot Organic farming is not obligatory but farming without chemicals is encouraged	Descriptive statistics Elements and motivation of gardening	Growing vegetables and consuming the harvest is key motivator for gardening Household composition Single: 10% With partner: 53% With children: 9% With partner and children: 27% Other: 1% Gardening duration (y) < 1: 9% 2–5: 22% 6–10: 28% 11–15: 12% 16–20: 4% > 20: 25%
Zoellner et al. 2012 [77]	The Dan River Region, VA, USA	Cross sectional survey	*n* = 87 youth, 67 parents Medically underserved area/population classification with high indices of poverty, low educational attainment, and health disparities *Characteristics* Unemployment in the region: 12.3–18.9%, well exceeding state (6.0%) and national (9.1%) averages *Children (n* = *87) n%* Mean age: 8.69 (SD 2.04) Female 42 (48.3) Male 45 (51.7) *Parents (n* = *67)* Mean age: 39.1 (9.16) Female 54 (80.6) Male 13 (19.4)	To understand factors impacting fruit, vegetable, and gardening behaviors	Youth (*n* = 129) and parents (*n* = 115) identified as potential participants and benefactors of future CG programming effort, enrolled in summer camp	Baseline data for understanding factors impacting gardening interests as well as fruit, vegetable, and gardening behaviors	Self-administered survey (44 items) on F&V intake, interest in gardening, height, weight Parent survey (58 questionnaire’s on availability of F&V, gardening attitudes) No adjustments reported	*Children (n* = *87)* Race/ethnicity Black: 47 (54.0) White: 36 (41.4) Hispanic: 2 ( 2.3) Other: 2 ( 2.3) Willingness to try F&V: 1.32 [SD 0.40) on a 2-point scale *Parents (n* = *67)* BMI Underweight: 1.7% Normal: 32.2% Overweight: 33.9% Obese: 32.2% Income ($) 0–19,999: 15.6% 20,000–49,999: 45.3% > 55,000: 39.1% Education High school diploma or less: 20.9% Some college, training, 2-year degree: 62.7% Bachelor’s degree: 7.5% Graduate school:9.0%
**Studies comparing gardeners with other groups including non-gardeners and home gardeners**								
Alaimo et al. 2010 [63]	Flint, MI, USA	Cross-sectional survey	Flint resident, aged ≥ 18 y who had lived at their current address for previous 12 months A final sample of 1,916 (63.6%) eligible respondents reached by phone agreed to be interviewed	To examine associations between participation in CG/beautification projects and neighborhood meetings with perceptions of social capital at both the individual (Objectives 1 and 2) and neighborhood levels (Objectives 3 and 4)	Part of Neighborhood Violence Prevention Collaborative (NVPC): a neighborhood development program Telephone survey administered in 2001 Random selection of phone numbers	Descriptive comparison to individuals not participating in community gardening or beautification projects	Descriptive only	Of 1916 individuals, *n* = 271 participated in community gardening or beautification projects (15.3 (SE: 1.0))% and *n* = 1224 did not participate Participants compared to non-participants: Age (y; mean ± SE): 40.7 ± 1.3 vs 43.5 ± 0.6 who didn’t participate Male: 45.7% vs. 43.3% Female: 54.3% vs. 56.7% White: 54.8% vs. 52.9% African American: 43.8% vs 42.7% Other: 1.4% vs. 4.4%
Christensen et al. 2019 [64]	Denmark, Copenhagen, Nordvest area High-density urban Multicultural	Cross sectional survey Statistics Denmark for neighborhood	150 gardeners at “Lersøgrøftens Integrationsbyhaver” (Urban Integration Gardens; UIG); *Characteristics* Age NR Sex NR	To examine UIG by assisting with challenged neighborhood and social capital	NR	Founded 2012 Modelled on urban renewal in neighboring area 150 garden plots shared equally among citizens born in versus outside of Denmark	SEP of gardeners vs non-gardeners Education Low/no ≥ degree Income Low-to-mid Mid- to high	*Education* *Low education* Gardeners: 10/75 (13%) Neighborhood: 10,558/17792(59%) *Degree or higher* Gardeners: 65/75 (87%) Neighborhood: 7234/17792(41%) Income *Low-to-mid* Gardeners: 42/75 (57%) Neighborhood: 26,433/17792(74%) *Mid-to-high* Gardeners: 32/75 (43%) Neighborhood: 9432/17792(26%)
Diekmann et al. 2020 [78]	USA, California, Santa Clara county	Cross-sectional survey	(1) Community Food Security (CFS) gardeners (*n* = 51) Female 84% Age (median): 49 (2) Home gardeners (*n* = 118) Female 81% Age (median): 57 (3) Community gardeners (*n* = 255) Female 61% Age (median): 58	To examine food insecurity according to 3 types of gardeners (1) low-income families offered the CFS gardening program, (2) home gardeners, (3) community gardeners	CFS gardeners recruited via local program. Home gardeners were a convenience sample of attendance at an annual garden market and via a listserv. Community gardeners sampled via stratified random sampling (4 geographic regions from which 10 gardens were randomly selected to receive an email invitation)	Not reported for the community gardeners	Characteristics of gardening groups compared using Chi- squared statistics. No adjustments	White race (1) 22% (2) 74% (3) 75% High school education (1) 30% (2) 0% (3) 1% Bachelor education (1) 32% (2) 83% (3) 84% Household income < $USD 7 K; 75-149 K; > 150 K (1) 88%; 12%; 0% (2) 17%; 44%; 38% (3) 28%; 33%; 39% Born overseas (1) 49% (2) 15% (3) 20% Home ownership (own; rent; other) (1) 40%; 52%; 8% (2) 93%; 7%; 0% (3) 77%; 18%; 5% Mean household size (1) 4.0 (2) 2.6 (3) 2.3 Food insecure (1) 39% (2) 3% (3) 10% Food assistance (1) 41% (2) 8% (3) 9%
Loopstra and Tarasuk 2013 [79]	Canada	Longitudinal	Total *n* = 501 families recruited to baseline study population (62% recruitment rate; *n* = 384 completed the follow-up interview, a return rate of 77%) - 359 families not using community garden program *N* = 12 did not provide a reason for not participating in a CG in previous 12 months Low-income population, disproportionate representation of immigrants and lone-parent families in the low-income population in Toronto. Very high prevalence of household food insecurity	To understand reasons for non-participation in a community garden, community kitchen program, or Good Food Box, in previous 12 months	Families with gross incomes at or below Statistics Canada’s mid- income adequacy category, living in subsidized and non-subsidized rental housing Door-to-door sampling in 12 neighborhoods randomly selected from the 23 “high poverty” census tracts in Toronto Structured oral interview with person in house- hold primarily responsible for household food purchases and management	No program. Study about characteristic of non-participation	Household income, demographics, food purchasing, household food insecurity; household participation in community gardens, community kitchens and the Good Food Box program Follow-up questionnaire: qualitative	Of the total sample *n* = 371 completed follow-up Lived < 2 km of CGs - YES: *n* = 245 (66.0%) - NO: *n* = 126 (34%) - Only 12 families at f/up (3.2%) indicated someone in household had participated in a CG Reasons for not participating in CG - 66.3% not accessible [28.4% lacked knowledge about how or where to participate; 24.2% not in neighborhood; 11.7% did not know what program was; 1.7% program capacity; 0.8% program eligibility; 0.6% program cost - 38.7% lack of fit [23.4% time; 11.7% interests; 3.3% needs; 3.1% health
Mwakiwa et al. 2018 [65]	Zimbabwe	Cross-sectional	Mainly from high-density suburbs, though some households from the medium and low-density suburbs also participated Each CG has 30 members with each member allocated 3 rows Each member averages 16 beds per row. CGs are grouped into clusters and each cluster consist of 2 to 4 CGs, in total 28 clusters	To examine the feasibility of community resource management in these gardens using a blend of econometrics and community resource management theory	Stratified sampling: household survey respondents were those still participating or discontinued. Random sample of 14 clusters (from 28). Then from each of the selected clusters, 10 households randomly selected. Total sample size = 140	93 fenced CGs, 1 ha each established	Interviews with key in- formants (i.e. housing and agriculture dept officers, and CG chairperson Binary logistic model (CG participation, yes/no); IV: household size and number of orphans; household size and density of suburb; number of orphans and density of suburb; and number of orphans and house ownership	From 136 households: - 26.5% no longer participating in CGs - 73.5% still participating Those who discontinued: 50% of households headed by males, 50% by females; older than those who continued (63 yrs vs 55 yrs) Those who continued: 56% headed by males and 44% by females Reasons for discontinuing - 41.6%, laborious and shortage of water - 19.4%, access to land elsewhere therefore no need for land in CGs - 16.7%, lack of land tenure security - households with less or no on-plot farming area have a higher probability of practicing community gardening than those with larger on-plot areas - Households in high densities are more likely to practice community gardening than households in the medium density suburbs
Roncarolo et al. 2015 [42]	Canada, Montreal	Cross-sectional study	Participants sampled from 16 traditional (e.g. food banks, *n* = 711) or 6 alternative (e.g. CGs) venues (*n* = 113) *Characteristics* Female: 55% Age: 52% aged 30–49 y	To compare outcomes between users of traditional versus alternative organizations	Sampled from food security organizations with ≥ 50 new members (traditional) or ≥ 30 new members (alternative)	Not precisely described but indicated as being organizations (gardens) that nurture solidarity, and have goals of reducing social inequalities	Household income (7 categories) Education (4 categories) Multilevel logistic regression to account for clustering by study site. Adjusted for sex, country of birth, marital status, employment, education, income and number of people in the household	Household income < $5 K OR_adjusted_ = reference $5 K- < 10 K OR_adjusted_ = 0.59 (0.23, 1.48) $10 K- < 15 K OR_adjusted_ = 0.89 (0.38, 2.09) $15 K- < 20 K OR_adjusted_ = 1.38 (0.46, 4.09) $20 K- < 30 K OR_adjusted_ = 2.51 (0.90, 6.95) $30 K- < 40 K OR_adjusted_ = 1.33 (0.38, 4.67) ≥ $40 K OR_adjusted_ = 4.51 (1.35, 15.11) Education < High school OR_adjusted_ = reference Secondary diploma OR_adjusted_ = 1.17 (0.58, 2.35) < Bachelor OR_adjusted_ = 1.56 (0.74, 3.29) ≥ Bachelor OR_adjusted_ = 3.76 (1.44, 9.79
Soga et al. 2017 [50]	Japan, Tokyo, Nerima district in central Tokyo	Cross-sectional survey	Gardeners (*n* = 165) vs non-gardeners (*n* = 167) *Characteristics* Gardeners: Male: 68.1% Age (mean ± SD): 62 ± 17y Non-gardeners: Male: 42% Age (mean ± SD): 61 ± 16y	To quantify effects of allotment gardening on physical, psychological and social health	Gardeners located by face-to-face recruitment at allotment gardens (90% response rate). Non-gardeners recruited via a letter sent to 1000 Nerima households (20% response rate)	NR	Household income, employment, smoking, drinking, and vegetable consumption	*(unadjusted)* Household income and smoking was similar, more gardeners than non-gardeners were retired (28% vs 18%), did not drink alcohol (31% vs 37%), and often consumed vegetables (54% vs 24%)
van den Berg et al. 2010 [51]	The Netherlands, “large cities”	Cross-sectional survey	Gardeners (*n* = 121) from 12 allotment gardens Non-gardener (*n* = 63) *Characteristics* Gardeners: Male: 53% Age (mean ± SD): 62 ± 12 y Non-gardeners: Male: 41% Age (mean ± SD): 56 ± 14 y	To directly compare the health, wellbeing and physical activity of allotment gardeners to that of controls without an allotment garden	Gardeners sent invitations to their home addresses Non-gardeners were responders living next to the home address of allotment gardeners	Ranged from residential parks, day-recreational parks and food production parks	Age, sex, employment, education, income, marital status, dependents, alcohol and smoking	Compared with non-gardeners, gardeners were older, more were male, retired (59% vs 33%), had fewer children living at home (13% vs 32%), consumed alcohol daily (62% vs 56$). However, there was little difference in the proportion married (62% for both), education levels (high school 38% vs 35%), income (< mode; 27% vs 29%), smoking (19% for both)

*Abbreviations*: *BMI* Body mass index, *CG* Community garden, *F&V* Fruit and vegetable, *NR* Not reported, *OR* Odds ratio, *SD* Standard deviation, *SE* Standard error, *SEP* socioeconomic position

### Quality of included studies

The quality assessment of the two RCTs have been included as Supplementary Table 2, and the quality assessments for other study designs are in Supplementary Table 3. Of the 34 non-randomized studies included in the review, only two were rated overall as having a low risk of bias. The most common problems were poor or no adjustment for confounding and the potential for selection bias. Deviation from any intended intervention was frequently unclear due to inadequate reporting, as was reporting of missing data.

### Discussion and conclusions

The results of this systematic review describe quantitative evidence from 53 studies (54 papers). The outcome with the largest amount of quantitative information was for fruit and vegetable intake, overall diet, nutrients or nutrition knowledge (*k* = 23 studies). Sixteen studies included health related outcomes, such as physical activity, BMI or blood pressure, and sixteen reported a diverse range of psychosocial outcomes such as happiness, stress and quality of life. Fewer studies reported community-related outcomes of gardeners (*k* = 7). Importantly, there were few studies located that were conducted in developing countries; the vast majority of studies reviewed here were from developed countries, particularly the United States.

Quite unexpectedly, 14 systematic reviews that had not been identified during the preliminary searches of databases were located. This is testament to how difficult this literature is to capture due to the varying terminology, breadth of outcomes examined, and places where this type of work has been published. Many of the other systematic reviews focus on specific content areas or a particular definition of gardening such as peri-urban agriculture, which has a different scope to our review. Where there was crossover, findings of the current review are somewhat similar to past reports though the current review is more up-to-date and suggests that ongoing (poor) quality of publications is proving difficult to shift.

For dietary outcomes, the results of the current review suggest that users of community gardens consume slightly more fruit and vegetables than non-users of community gardens, with little difference between findings of studies of low, moderate or serious risk of bias. Overall, the quality of the evidence is low with many studies at risk of selection bias and poor adjustment for confounding.

Harvesting fruit and vegetables from community gardens is typically seasonal and this may have influenced data collection, with few studies stating that had been taken into consideration. Of note is one publication indicating that community gardeners purchased more fruit and vegetables than other members of the community [41]. This might indicate that community gardeners are more interested in consuming fruits and vegetables than non-gardeners. Teasing apart the effects of community gardens from the effects of people who choose to use them is particularly challenging. The trial by Heilmayr and Friedman is neatly designed to tease apart the mechanism by which community gardens are purported to have effects, by using different comparison groups that focus on social contact, physical activity or outdoor exposure [34]. While this is a clever design for understanding the mechanisms, the RCT was underpowered and no effects on diet, activity or psychosocial outcomes were noted. Counter to expectations was that food security was consistently higher among community gardeners, as one study suggested highly food insecure participants were less likely to be involved in community gardens [42]. It is plausible to hypothesize that the community gardens may have made participants less food insecure or that gardens are not viewed by people experiencing food insecurity as a possible solution.

With respect to health outcomes, a wide variety of measures were reported in the included studies. It was common for articles to not report whether the more frequent measures such as BMI were self-reported or measured. Self-reported measures of BMI are often lower than measured BMI, and measured BMI is preferable particularly for pre/post designs, which might be vulnerable to outcome reporting bias. Nevertheless, studies indicated that community gardeners perceived themselves as having good to excellent health and as having lower odds of hypertension and overweight/obesity than non-gardeners. However the evidence was not consistent as one study [49] found no differences in physical activity, BMI, blood pressure and lung function between community gardeners and people in other active pursuits (such as home gardeners, walkers). This issue in particular, points to a need for careful consideration of who is being compared in each analysis, as well as the problem of self-selecting into active pursuits, such as community gardening, by healthy people.

Of the psychosocial outcomes, it is important to keep in mind the context. For example, psychosocial outcomes of community gardening from highly impoverished areas in low-income countries are not generalizable to high-income countries and vice versa. However, studies involving immigrants, refugees or culturally and linguistically diverse communities may be relevant. Among many potential benefits, the growth of culturally relevant produce may support resettlement. Gardeners tended to have more social contact and higher indicators of wellbeing than comparators, but again the body of evidence is both small and low in quality.

Of the seven studies reporting on indicators of community sentiment, gardeners rated neighborhood aesthetics and neighborhood attachment more highly than other members of their communities, and their civic participation is higher. Importantly, the current review did not distinguish between community gardens developed for the purposes of creating positive community sentiment or connection, and those gardens developed for the purposes of alleviating food security concerns. The differences in motivation for developing and participating in community gardens may well be important to consider as suggested in a review by Guitart and colleagues [80] and in empirical research from Trendov [81] and Bende and Nagy [82]. Despite motivational differences in community gardening, the current review suggests that the effect on social interactions and community connection appears to exist regardless. Once again, whether this finding is a result of community gardening or because people seeking social interactions self-select into gardening cannot be clearly delineated from the literature due to poor control of confounding and possible selection bias.

The aim to collect information on characteristics of community gardeners was made difficult by the majority of studies not comparing gardeners to either non-gardeners or to the general population. It would appear that community gardeners were generally older members of the community, with a higher proportion of retirees, and with more years of formal education. However, the samples included in individual studies are entirely dependent on the eligibility criteria (and research questions) of individual studies.

### Limitations

The limitations of the current review fall into two areas, those that arise as limitations of the studies included in the review and those that are limitations of the review processes itself. With respect to limitations of studies included in the review, there were no high quality well-powered RCTs and most of the evidence from observational research was rated as having a high risk of bias. The lack of randomized trials in this area is not surprising as it is difficult to randomize individuals to involvement (or not) in community gardens. Non-compliance within intervention and control groups would be problematic as some individuals in the treatment group would not interested in gardening, and some individuals in the control group would want to be gardening. This reflects the ‘problem’ of selection bias through self-selecting into desired activity (common to observational studies reviewed here). Other possibilities that could help elucidate the effects of community gardens could involve randomizing individuals as part of a prescription or treatment for health conditions, or randomizing entire communities to the implementation of a community garden though this would involve large commitments by councils and residents. Thus, the small amount of evidence from ‘gold standard’ RCTs will likely continue, and more attention should be paid to improving the quality of the observational evidence. Many studies had poor or no adjustment for confounding. Furthermore, careful attention needs to be paid to what is being compared in each study. For example, a comparison of food security outcomes from more advantaged community gardeners versus individuals accessing food banks could lead to over-estimates of the beneficial effects of gardens [42]. Even though such comparisons may be adjusted for confounders, it is unlikely that individuals are exchangeable on all other factors, and residual confounding is likely to be present. Thus, the evidence from individual observational studies are probably overly optimistic effects across all outcomes. Such challenges with research in the community gardens setting and the poor quality of evidence is unsurprising given the diversity of likely motivations for developing and participating in community gardens, the length of time needed to develop such gardens and then see any health or behavioral changes resulting from participation and the unique nature of each community garden and of the users themselves. Future research should not be dissuaded from investigating the benefits of community gardens, rather as much as practical, attention paid to the issues such as selection bias, adjustment for confounding and exchangeability.

Another potential limitation of the included studies is around external validity or in deciding whether the evidence from this review is applicable to other settings. Studies from low- or middle-income studies may not be directly applicable to high-income countries, and vice versa. However, there may also be external validity problems with the high-income country settings (where there is more evidence). For example, studies conducted in highly disadvantaged rural areas of the United States are not likely to be applicable to affluent areas of Europe or Asia, or to high-density living. Hence, the benefits observed in one setting may not be transferable to others.

Potential limitations from the systematic review process are predominantly around the inclusion of relevant literature and the scope of the outcomes. Databases that would have outcomes to inform the review were deliberately searched but no grey literature was searched and it is possible that potentially relevant studies were missed. Finding all sources of grey literature would be unrealistic for an academic review of this nature. If the results of grey and unpublished literature differed from the published literature, the current paper may have a potentially biased view of evidence. No formal tests of the potential for (positive) publication bias, were undertaken as the outcomes of studies were too disparate. As mentioned earlier, the literature in this field is published across many areas and there are many different terms used to reflect conceptualizations of ‘community gardens’. This became apparent during the search and screening processes. Potentially negative outcomes such as community gardens conducted in areas of poor or contaminated soil quality were also not considered. Although the search strategy located such articles, these studies were out of scope.

## Conclusions

In conclusion, the results of the studies included in this review indicate that community gardeners tend to consume more fruit and vegetables, are healthier and participate in civic settings more frequently than non-gardeners. However, the observational evidence that involves selected populations have poor (often no) adjustment for confounding, are at risk of bias. Thus, although the evidence is positive for all outcomes, the potential for bias is sufficiently high that the findings are likely to be overly optimistic effects of community gardens.

## Supplementary Information

**Additional file 1:** **Supplementary Table 1.** Search terms for each database. Table 1a PubMed search for studies on community gardens. Table 1b PsycINFO search for studies on community gardens. Table 1a Web of Science search for studies on community gardens. Table 1d EBSCOhost database searching for studies on community gardens. e CAB Abstracts search for studies on community gardens. Table 1f Summary of database searches for studies on community gardens. **Supplementary Table 2.** Quality assessment of the RCTs using the Cochrane Risk of Bias Assessment Tool (14). **Supplementary Table 3.** Quality assessment for included articles using the ROBINS-I Risk of Bias Assessment Tool (15).

## Data Availability

All data generated and analysed during this study are included in this published article.
