# Supplementary material for: Community gardens and their effects on diet, health, psychosocial and community outcomes: a systematic review
Source: BMC Public Health. 2022 Jun 23;22:1247. doi: 10.1186/s12889-022-13591-1 (PMC9229094; doi:10.1186/s12889-022-13591-1)
Supplement: Supplementary file 1 — Additional file 1: Supplementary Table 1. Search terms for each database. Table 1a PubMed search for studies on community gardens. Table 1b PsycINFO search for studies on community gardens. Table 1a Web of Science search for studies on community gardens. Table 1d EBSCOhost database searching for studies on community gardens. e CAB Abstracts search for studies on community gardens. Table 1f Summary of database searches for studies on community gardens. Supplementary Table 2. Quality assessment of the RCTs using the Cochrane Risk of Bias Assessment Tool (14). Supplementary Table 3. Quality assessment for included articles using the ROBINS-I Risk of Bias Assessment Tool (15). [file 12889_2022_13591_MOESM1_ESM.docx]

**Supplementary Table 1**: Search terms for each database

**Table 1a**: PubMed search for studies on community gardens

|  | Search terms | Number of hits |
| --- | --- | --- |
| 1 | ("Gardening"[Mesh]) OR ("Gardens"[Mesh]) | 1,202 |
| 2 | community garden* OR community vegetable garden* OR vegetable garden* OR veggie garden* OR vegie* garden OR vegetable garden* OR kitchen garden OR urban agriculture OR neighbourhood gard* OR neighbourhood gard* | 10,465 |
| 3 | #1 OR #2 | 11,305 |
| 4 | Limit #3 to studies in humans | 3,735 |
| 5 | Limit #4 to English language articles | 3,529 |
| 6 | Limit #5 to the following publication categories   - Clinical trial - Controlled clinical trial - Evaluation study - Journal article - Observational study - Pragmatic clinical trial - Randomized controlled trial | 3,443 |
| 7 | pathogen* OR dengue* OR virus OR viral OR wastewater OR waste water OR water quality OR soil OR wetland* OR heavy metal* OR dust OR microb* OR pollut* OR contamina* OR pesticide* OR phthalat* OR airborne particulate OR air borne particulates OR livestock OR herbivore* OR photosynth* OR gestational OR gestation OR fertili* OR crash* OR collision* OR cholera* OR schistosom* OR malaria* OR leptospir* OR schoolyard OR school yard OR day care OR child-rear* OR child rear* OR mammal* OR dominance OR hunt* OR moral OR justice OR jail* OR prison* OR soccer OR ancient OR knockdown OR knockout OR alcohol* | 7,191,016 |
| 8 | #6 NOT #7 | 1,671 |

**Date of last PubMed search: 4^th^ Dec 2020**

**Table 1b**: PsycINFO search for studies on community gardens

|  | Search terms | Number of hits |
| --- | --- | --- |
| 1 | Garden$ | 3,037 |
| 2 | urban agriculture | 31 |
| 3 | neighb$ OR community | 301,470 |
| 4 | 1 OR 2 | 3,058 |
| 5 | 3 AND 4 | 587 |
| 6 | Limit #5 to studies in humans | 553 |
| 7 | Limit #6 to English language articles | 547 |
| 8 | Limit #7 to the following publication categories   - peer-reviewed journal | 310 |
| 9 | Pathogen$ OR dengue$ OR virus OR viral OR wastewater OR waste water OR water quality OR soil OR wetland$ OR heavy metal$ OR dust OR microb$ OR pollut$ OR contamina$ OR pesticide$ OR phthalate$ OR airborne particulate OR air borne particulates OR livestock OR herbivore$ OR Photosynth$ OR gestational OR gestation OR fertili$ OR crash$ OR collision$ OR cholera$ OR schistosom$ OR malaria$ OR leptospir$ OR schoolyard OR school yard OR day care OR child-rear$ OR child rear$ OR mammal$ OR dominance OR hunt$ OR moral OR justice OR jail$ OR prison$ OR soccer OR ancient OR knockdown OR knockout OR alcohol$ | 473,727 |
| 10 | #8 NOT #9 | 284 |

**Date of last PsycINFO search: 4^th^ Dec 2020**

**Table 1c**: Web of Science search for studies on community gardens

|  | Search terms | Number of hits |
| --- | --- | --- |
| 1 | TS=(garden* OR urban agriculture) | 65,221 |
| 2 | TS=(neighb* OR community) | 1,736,196 |
| 3 | #1 and #2 | 7,835 |
| 4 | Limit #3 to English | 7,436 |
| 5 | Limit #4 to Articles | 6,283 |
| 6 | Limit #9 to Web of Science Core Collection: Citation Indexes   - Social Sciences Citation Index (SSCI) --1900-present - Arts & Humanities Citation Index (A&HCI) --1975-present - Conference Proceedings Citation Index- Social Science & Humanities (CPCI-SSH) --1990-present - Emerging Sources Citation Index (ESCI) --2015-present   (#5) AND LANGUAGE: (English) AND DOCUMENT TYPES: (Article)  Indexes=SSCI, A&HCI, CPCI-SSH, ESCI Timespan=All years | 3,130 |
| 7 | TS=(pathogen* OR dengue* OR virus OR viral OR wastewater OR waste water OR water quality OR soil OR wetland* OR heavy metal* OR dust OR microb* OR pollut* OR contamina* OR pesticide* OR phthalat* OR airborne particulate OR air borne particulates OR livestock OR herbivore* OR photosynth*) | 687,326 |
| 8 | TS=(gestational OR gestation OR fertili* OR crash* OR collision* OR cholera* OR schistosom* OR malaria* OR leptospir* OR schoolyard OR school yard OR day care OR child-rear* OR child rear* OR mammal* OR dominance OR hunt* OR moral OR justice OR jail* OR prison* OR soccer OR ancient OR knockdown OR knockout OR alcohol*) AND LANGUAGE: (English) AND DOCUMENT TYPES: (Article) | 746,423 |
| 9 | #6 NOT (#7 OR #8) | 2,368 |
| 10 | Web of Science categories   - Agriculture, Multidisciplinary - Anthropology - Behavioral Sciences - Ecology - Environmental Studies - Geography - Green & Sustainable Science & Technology - Horticulture - Integrative & Complementary Medicine - Medicine, Research & Experimental - Multidisciplinary Sciences - Nutrition & Dietetics - Primary Health Care - Psychology - Public, Environmental & Occupational Health - Regional & Urban Planning - Social Sciences, Interdisciplinary - Sociology - Sport Sciences - Urban Studies | 2,872,535 |
| 11 | #9 AND #10 | 1,575 |

**Date of last Web of Science search: 4^th^ Dec 2020**

**Table 1d**: EBSCOhost database searching for studies on community gardens

|  | Search terms | Number of studies identified |
| --- | --- | --- |
| 1 | "urban land use " OR “vertical farming” OR "agriculture" OR "gardens" OR “urban gardening” OR "community gardens" | 594,520 |
| 2 | "nutrition" OR "vegetable farming" OR "community kitchens" OR “vegetable gardening” OR “neighbo?rhood” | 478,864 |
| 3 | S1 AND S2 | 37,070 |
| 4 | Limit #3 to academic journal articles | 31,106 |
| 5 | Limit #4 to English | 30,792 |
| 6 | Refine #5 to the following subjects:   - Food security - Health behaviour - Nutrition - Neighbourhoods - Nutritional requirements - Food habits - Public health - Evaluation of human services programs - Food - Food consumption - Diet - Self-efficacy - Urban planning - Urban agriculture - Public health – united states - Body weight - Treatment effectiveness - Malnutrition | 1,121 |

**Date of last EBSCOhost database search: 7^th^ Dec 2020**

**Table 1e**: CAB Abstracts search for studies on community gardens

|  | Search terms | Number of studies identified |
| --- | --- | --- |
| 1 | TS=("urban agriculture " OR "gardening" OR "gardens") | 39,619 |
| 2 | TS=("community nutrition" OR "community programmes" OR "vegetable growing" OR "neighbo?rhood" OR "kitchen gardens") | 24,530 |
| 3 | #1 AND #2 | 33,056 |
| 4 | Limit #3 to journal articles | 25,995 |
| 5 | Limit #4 to English | 18,335 |
| 6 | Refine #5 to the following CABI codes   - Crop produce (944) - Field crops new march 2000 (928) - Human nutrition general (399) - Nutrition related disorders and therapeutic nutrition (454) - Human health and hygiene general (461) - Diet studies (222) - Food science and food products human (230) - Human health and the environment (269) | 3,275 |

**Date of last CAB Abstracts search: 4^th^ Dec 2020**

**Table 1f**: Summary of database searches for studies on community gardens

|  | Number of hits |
| --- | --- |
| PubMed | 1,671 |
| PsycINFO | 284 |
| Web of Science | 1,575 |
| EBSCOhost | 1,121 |
| CAB Abstracts | 3,275 |
| Subtotal | 7,926 |
| ***Total***  ***After duplicates removed*** | 7,355 |

**Supplementary Table 2**: Quality assessment of the RCTs using the Cochrane Risk of Bias Assessment Tool (14)

|  | Selection bias  Random sequence generation | Selection bias  Allocation concealment | Reporting bias  Selective reporting | Other sources of bias | Performance bias  Blinding (participants and personnel) | Detection bias  Blinding (outcome assessment) | Attrition bias  Incomplete outcome data | Overall quality |
| --- | --- | --- | --- | --- | --- | --- | --- | --- |
| Brown et al 2020 (25) | Serious | Unclear | Low | Low | High | Unclear | Serious | Poor |
| Heilmayr et al 2020 (35) | Unclear | Unclear | Unclear | Low | High | Unclear | Moderate | Poor |

**Supplementary Table 3**: Quality assessment for included articles using the ROBINS-I Risk of Bias Assessment Tool (15)

| Reference | Confounding | Selection | Classification of interventions | Deviations | Missing data | Measurement of Outcomes | Selection of the reported results | Overall risk of bias |
| --- | --- | --- | --- | --- | --- | --- | --- | --- |
| Alaimo et al 2008 (27) | Low | Low | Low | Low | Moderate | Low | Low | Low |
| Algert et al 2016 (30) | Serious | Serious | Low | Unclear | Unclear | Critical | Low | Serious |
| Barnidge et al 2013 (26) | Serious | Critical | Low | Low | Low | Critical | Low | Serious |
| Barnidge et al 2015 (32) | Low | Moderate | Low | Serious | Low | Serious | Low | Serious |
| Carney et al 2012 (28) | Serious | Serious | Low | Unclear | Low | Critical | Low | Critical |
| Castro et al 2013 (29) | Serious | Serious | Low | Unclear | Moderate | Critical | Low | Critical |
| Comstock et al 2010 (61) | Moderate | Moderate | Low | Moderate | Low | Low | Low | Moderate |
| Diekmann et al 2020 (78) | Serious | Moderate | Serious | Moderate | Moderate | Low | Low | Serious |
| De Marco et al 2016 (33) | Serious | Serious | Moderate | Moderate | Unclear | Low | Low | Serious |
| Gerber et al 2017 (56) | Serious | Serious | Low | Unclear | Low | Low | Low | Serious |
| Grier et al 2015 (57) | Serious | Serious | Low | Low | Low | Low | Low | Critical |
| Hartwig & Mason (34) | Serious | Moderate | Low | Low | Moderate | Low | Unclear | Critical |
| Hawkins et al 2011 (49) | Serous | Moderate | Serious | Moderate | Low | Low | Low | Serious |
| Hopkins & Holben 2018 (36) | Serious | Low | Low | Low | Unclear | Serious | Unclear | Critical |
| Kim 2017 (31) | Serious | Low | Low | Low | Unclear | Serious | Unclear | Critical |
| Koay & Dillon 2020 (58) | Moderate | Serious | Moderate | Moderate | Moderate | Low | Low | Serious |
| Litt et al. 2011 (37) | Low | Moderate | Low | Unclear | Low | Low | Low | Low |
| Litt et al 2015 (38) | Moderate | Low | Serious | Low | Low | Low | Low | Serious |
| Machida 2019 (39) | Low | Moderate | Moderate | Low | Unclear | Low | Unclear | Moderate |
| Mangadu et al 2017 (40) | Serious | Unclear | Serious | Unclear | Unclear | Moderate | Low | Serious |
| Martin et al 2017 (41) | Moderate | Serious | Low | Unclear | Low | Moderate | Moderate | Serious |
| Mourao et al 2019 (54) | Serious | Serious | Low | Unclear | Low | Low | Low | Serious |
| Roncarolo et al 2015 (42) | Moderate | Moderate | Low | Moderate | Low | Low | Low | Moderate |
| Schmidt & Vorster 1995 (43) | Serious | Unclear | Moderate | Low | Unclear | Low | Low | Moderate |
| Soga et al 2017 (50) | Low | Moderate | Low | Low | Low | Low | Low | Moderate |
| Spees et al 2016 (44) | Serious | Serious | Serious | Unclear | Unclear | Moderate | Low | Serious |
| Spliethoff et al 2016 (45) | Serious | Low | Low | Unclear | Low | Unclear | Unclear | Serious |
| Swami 2020 (59) | Serious | Low | Low | Moderate | Unclear | Low | Low | Serious |
| Tharrey et al 2020 (46) | Moderate | Low | Low | Low | Moderate | Low | Low | Moderate |
| Van den Berg et al 2010 (51) | Low | Moderate | Low | Low | Low | Low | Low | Moderate |
| Veen et al 2016 (47) | Serious | Moderate | Low | Low | Unclear | Low | Unclear | Critical |
| Weltin & Lavin 2012, Weltin 2013 (48, 52) | Moderate | Serious | Moderate | Serious | Moderate | Low | Serious | Serious |
| Young et al 2020 (60) | Low | Moderate | Low | Low | Low | Low | Low | Moderate |
| Zick et al. 2013 (53) | Low | Moderate | Low | Low | Unclear | Moderate | Low | Moderate |

Abbreviations: NA, not applicable because the study did not make any comparisons
